# A high fidelity approach to assembling the complex *Borrelia* genome

**DOI:** 10.1186/s12864-023-09500-4

**Published:** 2023-07-17

**Authors:** Sabrina Hepner, Konstantin Kuleshov, Ave Tooming-Kunderud, Nikolas Alig, Alexander Gofton, Sherwood Casjens, Robert E. Rollins, Alexandra Dangel, Evangelos Mourkas, Samuel K. Sheppard, Andreas Wieser, Johannes Hübner, Andreas Sing, Volker Fingerle, Gabriele Margos

**Affiliations:** 1 German National Reference Centre for Borrelia, Oberschleissheim, Germany; 2 Bavarian Health and Food Safety Authority, Oberschleissheim, Germany; 3 Central Research Institute of Epidemiology, Moscow, Russia; 4 Department of Biosciences, Norwegian Sequencing Centre at Centre for Ecological and Evolutionary Synthesis, University of Oslo, Oslo, Norway; 5 CSIRO, Health and Biosecurity, Canberra, ATC Australia; 6 Division of Microbiology and Immunology, Pathology Department, University of Utah School of Medicine, Salt Lake City, UT USA; 7 Institute of Avian Research “Vogelwarte Helgoland”, Wilhelmshaven, Germany; 8 Department of Biology, University of Oxford, Oxford, UK; 9 Medical Microbiology and Hospital Epidemiology, Max von Pettenkofer Institute, Faculty of Medicine, LMU Munich, Munich, Germany; 10 Division of Infectious Diseases and Tropical Medicine, LMU University Hospital, LMU Munich, Munich, Germany; 11 German Center for Infection Research (DZIF), partner site Munich, Munich, Germany; 12 Imunology, Infectious Disease and Pandemic Research IIP, Fraunhofer Institute for Translational Medicine and Pharmacology ITMP, Munich, Germany; 13 Dr. Von Hauner Children’s Hospital, LMU Munich, Munich, Germany

**Keywords:** *Borrelia burgdorferi*, Genomics, Plasmids, HiFi sequencing, De novo assembly, Genome reconstruction pipeline

## Abstract

**Background:**

Bacteria of the *Borrelia burgdorferi* sensu lato (s.l.) complex can cause Lyme borreliosis. Different *B. burgdorferi* s.l. genospecies vary in their host and vector associations and human pathogenicity but the genetic basis for these adaptations is unresolved and requires completed and reliable genomes for comparative analyses. The de novo assembly of a complete *Borrelia* genome is challenging due to the high levels of complexity, represented by a high number of circular and linear plasmids that are dynamic, showing mosaic structure and sequence homology. Previous work demonstrated that even advanced approaches, such as a combination of short-read and long-read data, might lead to incomplete plasmid reconstruction. Here, using recently developed high-fidelity (HiFi) PacBio sequencing, we explored strategies to obtain gap-free, complete and high quality *Borrelia* genome assemblies. Optimizing genome assembly, quality control and refinement steps, we critically appraised existing techniques to create a workflow that lead to improved genome reconstruction.

**Results:**

Despite the latest available technologies, stand-alone sequencing and assembly methods are insufficient for the generation of complete and high quality *Borrelia* genome assemblies. We developed a workflow pipeline for the de novo genome assembly for *Borrelia* using several types of sequence data and incorporating multiple assemblers to recover the complete genome including both circular and linear plasmid sequences.

**Conclusion:**

Our study demonstrates that, with HiFi data and an ensemble reconstruction pipeline with refinement steps, chromosomal and plasmid sequences can be fully resolved, even for complex genomes such as *Borrelia*. The presented pipeline may be of interest for the assembly of further complex microbial genomes.

**Supplementary Information:**

The online version contains supplementary material available at 10.1186/s12864-023-09500-4.

## Background

### Description of *B. burgdorferi* s.l. species complex

Lyme borreliosis (LB) is caused by several species of the *B. burgdorferi* sensu lato (s.l.) complex that includes 22 species with proposed or validly published names of which six are human pathogens: *Borrelia afzelii*, *B. garinii*, *B. burgdorferi* sensu stricto (s.s.), *B. spielmanii*, *B. bavariensis* and *B. mayonii* [1–4]. Bacteria in this complex are maintained in natural transmission cycles between tick vectors and various vertebrate, reservoir hosts [4–8]. Genospecies within the complex differ in human pathogenicity and virulence, and in their vector and/or host associations [9–13].

### The complex genome of *Borrelia*

Phenotype variation is reflected in genome complexity in this genus. Although relatively small (~ 1.5 Mb), the *Borrelia* genome it is highly complex and structurally unique compared to most bacteria [14–16]. The genome is highly fragmented consisting of a linear chromosome (ranging from 900 to 920 kb in size) and can have more than 20 circular and linear plasmids (ranging from 5 to 84 kb in size). Plasmids contribute about 40% to the whole genome content and currently 37 different LB *Borrelia* putative plasmid compatibility types are known [15–25].

The linear *Borrelia* chromosome and linear plasmids are highly unusual in bacteria and maintained by covalently-closed hairpin telomeres that terminate the linear replicons [16, 26–28]. The chromosome carries mostly housekeeping genes and shows conserved syntheny, while plasmid presence and gene content can show high variability [17, 18, 25, 29]. Apart from two conserved plasmids (cp26 and lp54) [16, 19, 20], the other plasmids are more variable and show rearrangements between and within plasmid types and even plasmid fusions (all together known as mosaic structure). This results in differences in gene content of plasmids belonging to the same plasmid type [17–19, 29, 30]. Consequently, two isolates with an identical repertoire of plasmid types can differ in gene content and identical genes may be present on different plasmid types.

High variability in plasmids suggests that they play a major role in maintaining the bacteria in natural transmission cycles, and indeed plasmid encoded genes are important for host and vector interaction [20, 25, 29, 31–35]. Complete, reliable and error-free plasmid sequences are essential for comparative genomics studies aiming to investigate molecular adaptation factors by gene absence vs presence studies, as unfinished genomes can lead to missed gene calls [36, 37]. Due to the high complexity, mosaic structure and homology between plasmids, assembly of the *Borrelia* plasmid sequences poses a formidable challenge to sequencing technologies and assembly tools.

### The state-of-the-art in *Borrelia* sequencing

Some bacteria possess complex genomes, making the construction of finished genome assemblies challenging, especially if there is no completed reference genome. Previous *Borrelia* genome sequencing projects have demonstrated that the use of short-read NGS sequencing alone, is insufficient for *Borrelia* plasmid assembly [15, 29, 38–43]. Long-read sequencing technologies have greatly improved the complete reconstruction of plasmids and chromosome including challenging areas [21, 44, 45]; however, incomplete plasmid sequences are still not uncommon [22, 46]. Further studies showed that a combination of noisy long-read (as scaffold) and accurate short-read (for error correction) data led to improvements in genome assembly while Pacific Biosciences (PacBio) seemed the most suitable long-read technology for *Borrelia* in comparison to Oxford Nanopore technology (ONT) [15, 23, 42, 47, 48]. These improvements were promising in terms of completing *Borrelia* genomes. Nonetheless, incomplete, non-assembled and incorrectly fused plasmids have also been reported with this approach [29, 47, 48]. Recently, PacBio introduced highly accurate long high-fidelity (HiFi) reads that are produced using circular consensus sequencing (CCS). HiFi long-read data is claimed to be similar accurate as short reads, which would represent a significant advancement in the long read technology [49, 50].

### The high fidelity approach for *Borrelia* genome reconstruction

Here, we analyze whether the recent introduced PacBio HiFi data and newly developed assembly tools could solve the *Borrelia* plasmid assembly problems. For this, we sequenced and assembled three *B. burgdorferi* s.l. isolates belonging to the species *B. bavariensis*, *B. garinii* and *B. valaisiana* by using three different assemblers (microbial, IPA and HiCanu). The genome reconstruction, including quality check (QC) and refinement steps, is shown in detail for these three samples. Initial data from a further 24 isolates support the findings presented for the three isolates. Here, we present an ensemble reconstruction pipeline that enables the complete de novo reconstruction of the complex *Borrelia* genome.

## Results

### Ensemble pipeline for complete *Borrelia* genome reconstruction

**Fig. 1:**
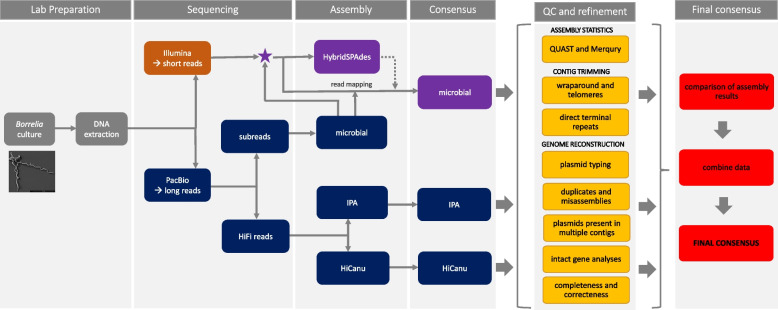
Schematic overview of the ensemble pipeline for *Borrelia* genome reconstruction established in this study. Lab preparation steps are indicated in grey. Data based on PacBio sequencing is shown in dark blue, data based on Illumina sequencing is shown in orange. A combination of PacBio and Illumina data is colored purple. QC and refinement steps are shown in yellow and the steps to generate the final consensus are shown in red

In this study we show that the reconstruction of a complete *Borrelia* genome is possible using different sequencing technologies and assembly strategies, including several manual curation steps and merging of the different assemblies. These steps can be summarized in an ensemble pipeline that enables the reconstruction of complete *Borrelia* genomes. An overview of the pipeline is shown in Fig. 1 and will be summarized below (see material and methods for details).

*Borrelia* strains were cultured and DNA was extracted (grey in Fig. 1). Sequencing was performed using PacBio SMRT long-read sequencing technology (blue in Fig. 1) and Illumina short-read technology (orange in Fig. 1). The PacBio long-read sequencing resulted in two datasets: PacBio subreads and PacBio HiFi (CCS with minimum number of 3 passes and minimum accuracy of 0.99) reads. For PacBio subreads the PacBio microbial assembler was used to generate assemblies, for the HiFi reads the PacBio Improved Phase Assembler (IPA) and the HiCanu assembler were used. The microbial assembly is based on low accuracy PacBio subreads and was polished using highly accurate Illumina reads. In addition, a hybridSPAdes assembly was performed on the Illumina data and PacBio microbial contigs. In the case of poor quality or incompleteness of contigs generated via the microbial assembler, it was replaced by, or concatenated with, the hybridSPAdes contig. The consensus of the microbial assembly may therefore result from a combination of Illumina and PacBio subread data (purple in Fig. 1). In contrast, assemblies based on the highly accurate PacBio HiFi reads were polished using HiFi reads instead of Illumina data. Therefore, the consensus of the IPA and HiCanu contigs is only based on PacBio HiFi data. Afterwards, quality control and refinement steps were conducted for the microbial, IPA and HiCanu consensi (yellow in Fig. 1). Finally, the assembly results were manually compared regarding correctness and completeness and combined to generate the final consensus representing a completed *Borrelia* genome (red in Fig. 1).

In the following, the results of the QC and refinement steps as well as the generation of the final consensus are described in detail.

### QC and refinement steps

#### Assembly statistics and quality

Assembly statistics and quality results (number of contigs, largest contig, total length, N50, L50 and completeness) determined using QUAST and Merqury are shown in Table S1.

We observed a trend of lowest contig number (potentially indicating high quality) resulting from the IPA assembler and the highest contig numbers (potentially indicating low quality) resulting from the HiCanu assembler, while no trend was noticed in the case of the microbial assembler (Table S1). Although the IPA assembler seemed promising due to low contig numbers, we observed typically shortest contigs, minimum total length, low N50, high L50 and the lowest degree of assembly completeness. Further analyses revealed that it tended to have more incompletely assembled genome elements. This indicated a limited suitability of the IPA assembler for *Borrelia* genome reconstruction. In contrast, HiCanu assemblies showed the highest contig number, it typically generated the largest contigs, maximum total length, high N50, low L50 and the highest degree of assembly completeness, which indicated that it performed very well. Further analyses confirmed the high quality of the HiCanu assemblies and the high number of contigs could be explained due to duplicates (more information is shown in section “Genome reconstruction from different assemblies”). The microbial assembler also resulted in good assembly statistics and performed nearly as well as the HiCanu assembler.

#### Contig trimming

**Fig. 2:**
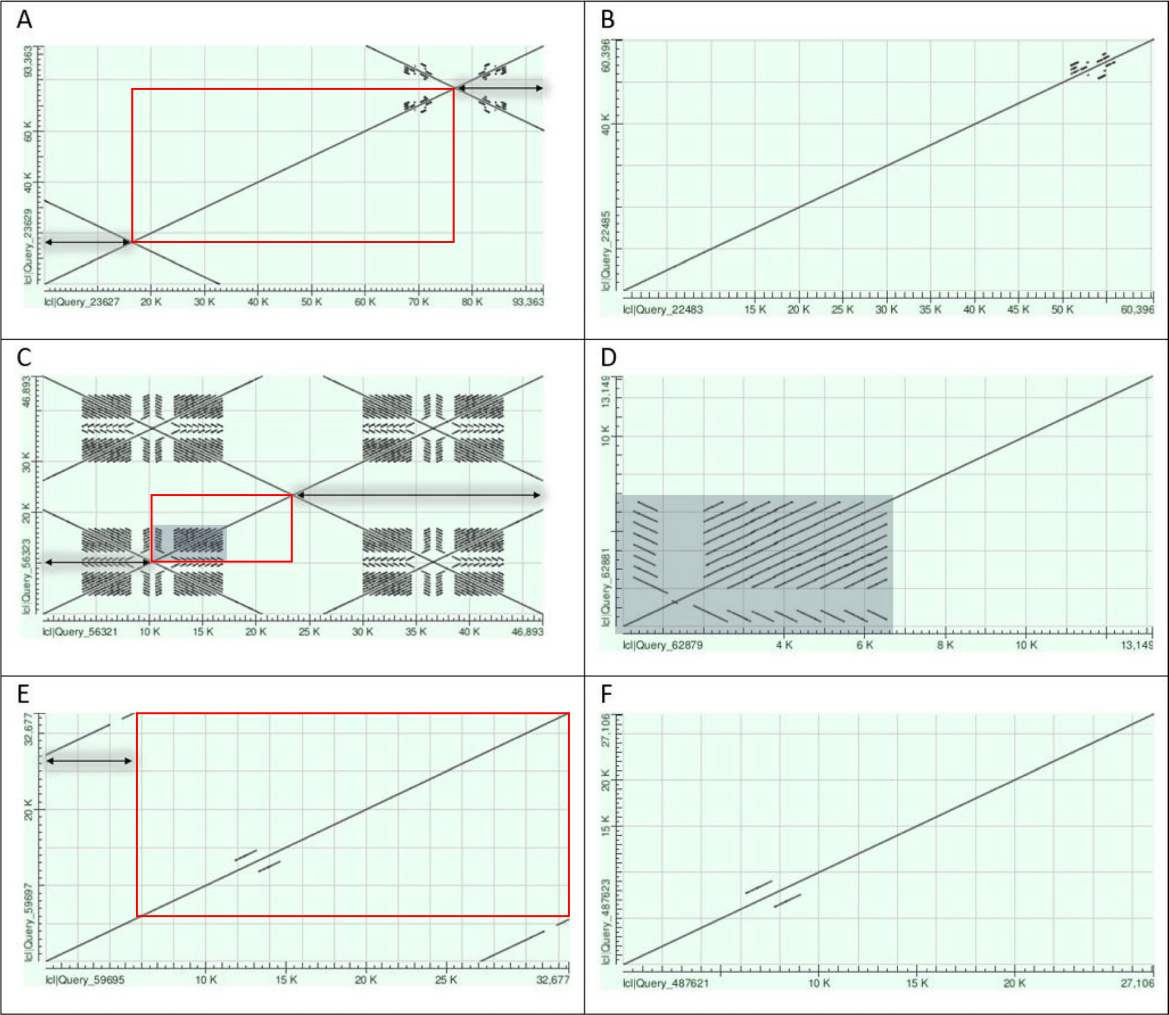
Dot plot examples before (left) and after (right) contig trimming. Wraparound and terminal direct repeats that need to be trimmed are indicated by a black arrow. The remaining part after trimming is indicated by a red box. Dot plot of PBaeII lp54 (contig ctg.s2.000000F of the microbial assembly) untrimmed (**A**) and trimmed (**B**). Dot plot of PBaeII lp28-8 (contig ctg.s2.000004F of the microbial assembly) untrimmed (**C**) and trimmed (**D**). The region of the *vls* locus is indicated by a gray filled box. Dot plot of PBaeII cp26 (contig tig00000016 of the HiCanu assembly) untrimmed (**E**) and trimmed (**F**). Dot plots were generated using the web-based NCBI-BLASTN [51]

PacBio contigs often contain reads that wrap around the hairpin ends of linear elements to generate long inverted repeats. If such wraparounds in untrimmed contigs contain the telomere consensus sequence, TAGTATA typically 14 bp from the center of the wraparound inverted repeat (to be described in more detail in a subsequent publication), the wraparound is considered to indicate the presence of a telomere on a linear replicon. Circular plasmid contigs, on the other hand, typically show terminal direct repeats due to their circular and continuous structure. The wraparounds and terminal direct repeats need to be trimmed off to generate a correct final sequence, and dot plot analyses were used to identify their presence. Figure 2 shows several examples of dot plots of contigs with wraparounds (linear genome elements) and terminal direct repeats (circular) before and after trimming.

Figure 2A and B show the two dot plots of PBaeII lp54 (contig ctg.s2.000000F of the microbial assembly) before and after trimming, respectively. The untrimmed contig contains long wraparounds of several thousand bases at the left end (1 bp – 16,417 bp) and right end (76,814 bp – 93,363 bp) of the contig (wraparounds are indicated by black arrows in Fig. 2A). After trimming both ends, the dot plot forms one continuous straight line from the beginning to the end of the contig (Fig. 2B). If wraparounds or terminal direct repeats are not present, this may be an indication of incompleteness of the linear or circular genome element, respectively.

As *Borrelia* plasmids may contain repetitive sequences or have high similarity stretches within the contig, the dot plots may show similarity lines that should not be trimmed. An example is shown in Fig. 2C and D, which depicts the dot plots of PBaeII lp28-8 (contig ctg.s2.000004F of the microbial assembly) before and after trimming. The lp28 plasmid family may contain the *vls* locus including silent cassettes (repetitive sequences) adjacent to the expression site, which can be observed in the dot plot (gray filled box in Fig. 2C and D). Figure 2C shows the untrimmed contig with wraparounds on the left side (1 bp – 10,298 bp) and right side (23,448 bp – 46,893 bp) of the contig. After trimming (Fig. 2D) the dot plot still shows the *vls* silent cassettes that produce the similarity pattern but should not be trimmed.

In contrast to the linear genome elements, contigs of the circular plasmids may contain terminal direct repeats. In this case, the first part of the contig corresponds to the end of the contig, which shows that the contig should be circularized and that the plasmid is complete. Figure 2E shows the dot plot of PBaeII cp26 (contig tig00000016, HiCanu assembly) with terminal direct repeats at the ends (1 bp – 5,571 bp overlapping 27,106 bp – 32,677 bp). In this case, the contig should only be trimmed at one side (e.g. 1 bp – 5,571 bp, black arrow in Fig. 2E). Figure 2F shows the dot plot of PBaeII cp26 after trimming.

Dot plots for all the contigs of the microbial, IPA and HiCanu assembler for sample PBaeII are shown in additional information (Figure S1 – Figure S3).

#### Genome reconstruction from different assemblies

For the genome reconstruction, every contig of the microbial, IPA and HiCanu assembly was analyzed in detail, including plasmid typing, identification of duplicates, misassemblies and genome elements present in multiple contigs. To include biological and evolutional information about *Borrelia*, plasmids were typed according to their paralogous gene family PFam32. The family contains also PFam49, PFam50 and PFam57/62 that refer to previously described gene families in *B. burgdorferi* s.s. B31 [17–20, 25]. The summary of these analyses for the representative isolate *B. bavariensis* PBaeII is shown in Table 1.

**Table 1:** Single contig analyses of the microbial, IPA and HiCanu assembly of PBaeII

**Assembler**	**Contig**	**Length**	**Length trimmed**	**PFam32 locus**	**Comment**	**Genome element**	**Consensus length**
**microbial**	ctg.s1.000000F	885916	885916	-	concatenate to chromosome	chromosome	905911
	ctg.s2.000000F	93363	60396	lp54	-	lp54	60397
	ctg.s2.000001F	55952	41416	cp32-3	concatenate with hyrbidSPAdes	cp32-3+lp25_incomplete	46802
	ctg.s2.000002F	39059	19532	-	concatenate to chromosome	-	-
	ctg.s2.000003F	43315	36788	lp28-4+cp32-1	-	lp28-4+cp32-1 _incomplete	36786
	ctg.s2.000004F	46893	13149	lp28-8	-	lp28-8	13162
	ctg.s2.000005F	34879	19448	-	concatenate to chromosome	-	-
	ctg.s2.000006F	41548	24153	lp28-3	-	lp28-3	24153
	ctg.s2.000007F	50489	21395	lp36	-	lp36	21397
	ctg.s2.000008F	26711	15157	-	-	lp28-7_incomplete	15163
	ctg.s2.000009F	35548	17911	-	-	lp17_incomplete	17912
	ctg.s2.10arro	27107	27107	cp26	-	cp26	27107
	ctg.s2.12arro	21095	21095	cp32-4	-	cp32-4	21095
	ctg.s2.14arro	29941	29941	cp32-5	-	cp32-5	29944
**IPA**	ctg.000000F	930217	905913	-	-	chromosome	905913
	ctg.000001F	70596	56122	lp54	-	lp54_incomplete	56122
	ctg.000002F	39819	39819	cp32-3+lp25	-	cp32-3+lp25_incomplete	39819
	ctg.000003F	28849	28849	cp32-5	-	cp32-5_incomplete	28849
	ctg.000004F	29068	14702	-	-	lp28-3_incomplete	14702
	ctg.000005F	31957	18069	lp36	-	lp36_incomplete	18069
	ctg.000006F	28109	28109	lp28-4+cp32-1	-	lp28-4+cp32-1 _incomplete	28109
	ctg.000007F	33304	13160	lp28-8	-	lp28-8	13160
	ctg.000008F	13940	8722	-	duplicate and misassembly	-	-
	ctg.000009F	16360	16360	-	-	lp17_incomplete	16360
	ctg.000010F	14120	14120	-	-	lp28-7_incomplete	14120
	ctg.11	27107	27107	cp26	-	cp26	27107
	ctg.13	21099	21099	cp32-4	-	cp32-4	21099
**HiCanu**	tig00000001	930187	905912	-	-	chromosome	905912
	tig00000003	21178	21178	-	duplicate of chromosome	-	-
	tig00000004	19699	19699	-	duplicate of chromosome	-	-
	tig00000005	15112	15112	-	duplicate of chromosome	-	-
	tig00000006	14495	14495	-	duplicate of chromosome	-	-
	tig00000008	23702	23702	-	duplicate of chromosome	-	-
	tig00000009	84366	60397	lp54	-	lp54	60397
	tig00000010	80586	54929	cp32-3+lp25	-	cp32-3+lp25	54929
	tig00000011	75901	50735	lp28-4+cp32-1	-	lp28-4+cp32-1	50735
	tig00000012	50555	28286	lp28-7	-	lp28-7	28286
	tig00000014	29941	29941	cp32-5	-	cp32-5	29941
	tig00000015	25964	21829	cp32-4	-	cp32-4	21829
	tig00000016	32677	27106	cp26	-	cp26	27106
	tig00000018	46995	21394	lp36	-	lp36	21394
	tig00000019	26898	13496	lp17	concatenate to lp17	lp17	24961
	tig00000020	9677	9677	-	duplicate of lp17	-	-
	tig00000021	9099	9099	-	duplicate of lp17	-	-
	tig00000022	39099	19609	-	concatenate to lp17	-	-
	tig00000023	28698	14353	-	concatenate to lp28-3	lp28-3	24137
	tig00000024	30200	16799	lp28-3	concatenate to lp28-3	-	-
	tig00000025	8619	8619	-	duplicate of lp28-3	-	-

The microbial assembly of PBaeII resulted in 14 contigs with five contigs lacking the PFam32 locus (Table 1). Three of these contigs (ctg.s1.000000F, ctg.s2.000002F and ctg.s2.000005F) were part of the chromosome which had overlapping sequences and were concatenated (overhangs were attached to scaffold contig ctg.s1.000000F). The other two contigs were incompletely assembled plasmids lacking the portion where the PFam32 locus would be located (ctg.s2.000008F and ctg.s2.000009F). Due to the lack of the PFam32 locus, the type of the incomplete assembled plasmid could not be determined and were only revealed by comparison with the other assembly results (ctg.s2.000008F: lp28-7_incomplete, ctg.s2.000009F: lp17_incomplete). Contig ctg.s2.000001F contained a cp32-3 type PFam32 locus but further analyses and comparison with the results of the other assemblers (IPA and HiCanu) showed that the plasmid was apparently a cp32-3 + lp25 fusion plasmid that was incomplete and therefore only contained one PFam32 locus.

The IPA assembler generated 13 PBaeII contigs and five of them did not contain the PFam32 locus (Table 1). One of these contigs represented the chromosome (ctg.000000F) and three were incomplete plasmids where the type was only revealed by comparison with the other assembly results (ctg.000004F: lp28-3_incomplete, ctg.000009F: lp17_incomplete, ctg.000010F: lp28-7_incomplete). Contig ctg.000008F was a misassembled duplicate and was deleted.

The HiCanu assembler produced 21 PBaeII contigs where 11 did not have the PFam32 locus (Table 1). One of the contigs represented the 906 kb chromosome (tig00000001) and five contigs were duplicates of portions of the chromosome (tig00000003 (21 kb), tig00000004 (20 kb), tig00000005 (15 kb), tig00000006 (14 kb), tig00000008 (24 kb) with identities of 99.87%, 99.94%, 99.93%, 99.81% and 99.78%, respectively) and were deleted. Two contigs (tig00000019 and tig00000022) had overlapping sequences where only one contig contained the PFam32 locus (tig00000019) and both contigs were concatenated to lp17. Further two contigs tig00000020 (10 kb) and tig00000021 (9 kb) did not possess the PFam32 locus, and were duplicates of portions of the concatenated lp17 (25 kb) with identities of 99.98% and 100%, respectively. As such, these contigs were deleted. Similarly, contig tig00000023 and tig00000024 showed overlapping sequences where only the latter carried the PFam32 locus. Both contigs were combined to form the lp28-3 plasmid. The contig tig00000025 (9 kb) was a duplicate of a portion of the concatenated lp28-3 (24 kb) with an identity of 99.71% and was deleted.

**Fig. 3:**
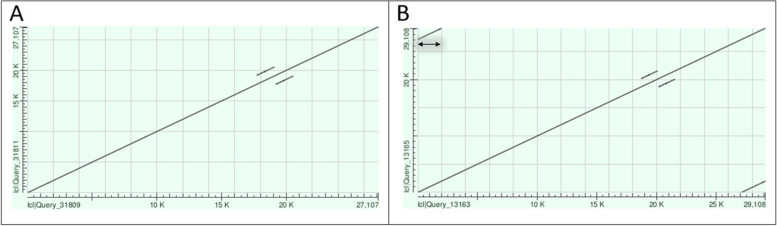
Dot plot of contig ctg.s2.10 (cp26) of the microbial assembly of PBaeII without terminal direct repeats (left) and containing terminal direct repeats after extension (right). In the left panel (**A**) is the untrimmed contig that does not show terminal direct repeats, in the right panel (**B**) is the extended contig which contains the overlapping region (1 bp – 2,000 bp overlaps 27,108 bp – 29,108 bp). Therefore, the plasmid can be considered as circular and complete. Dot plots were generated using the web-based NCBI-BLASTN [51]

The genome elements were analyzed for the number of intact genes and completeness. The latter is indicated by the presence of wraparound telomere sequences at both ends of linear replicons or terminal direct repeat in circular plasmids in untrimmed contigs and by the presence of PFam32 or related partition gene loci. If a circular plasmid did not show terminal direct repeats, the sequence was extended and reanalyzed by dot plot generation (for details see materials and methods). Figure 3 shows the dot plots of contig ctg.s2.10 (cp26) of the microbial assembly of PBaeII, which was considered complete as direct terminal repeats were found after sequence extension.

Plasmids that were reconstructed by concatenation of overlapping contigs were reanalyzed for the presence of wraparound and terminal direct repeats and were given a final trim (see section *“*Contig trimming*”*).

It must be emphasized that the analysis steps of "contig trimming" and "genome reconstruction" are partially intertwined and are dependent on one another, since there is no fixed order for the analysis.

### Generation of final consensus

#### Comparison of assembly results

**Fig. 4:**
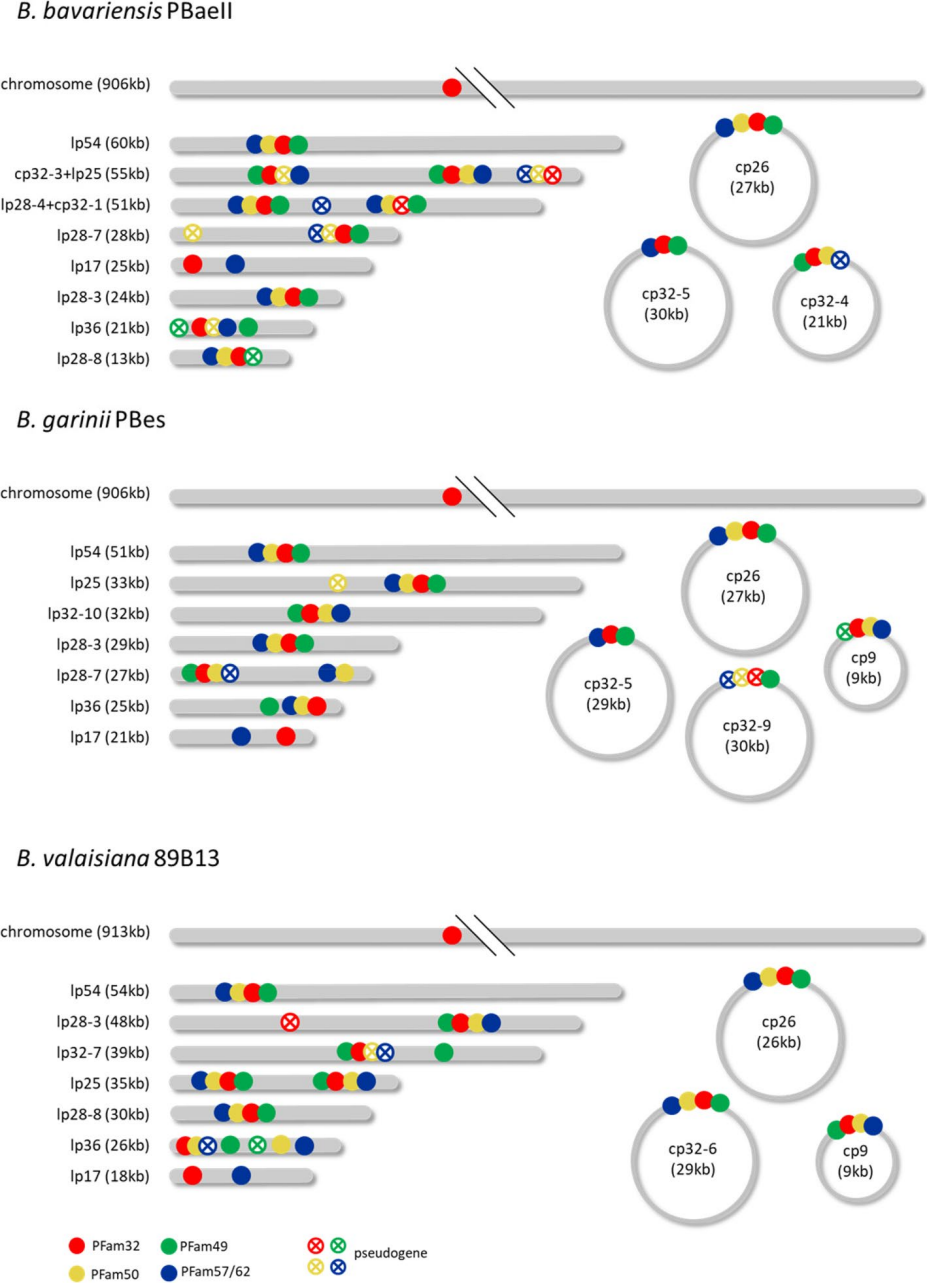
Schematic visualization of the genome elements of PBaeII, PBes and 89B13. Partitioning genes are shown as colored dots (PFam32: red, PFam49: green, PFam50: yellow, PFam57/62: blue). Intact genes are shown as filled dots, pseudogenes are shown as unfilled points with a cross. Intact genes and pseudogenes were defined using the NCBI annotator PGAP [52]

For each of the three representative isolates (PBaeII, PBes and 89B13) the polished assembly results produced by the three assemblers (microbial, IPA and HiCanu) are shown in Fig. 4 and Table 2. Further detailed information can be found in the additional information (Table S2).

**Table 2:**
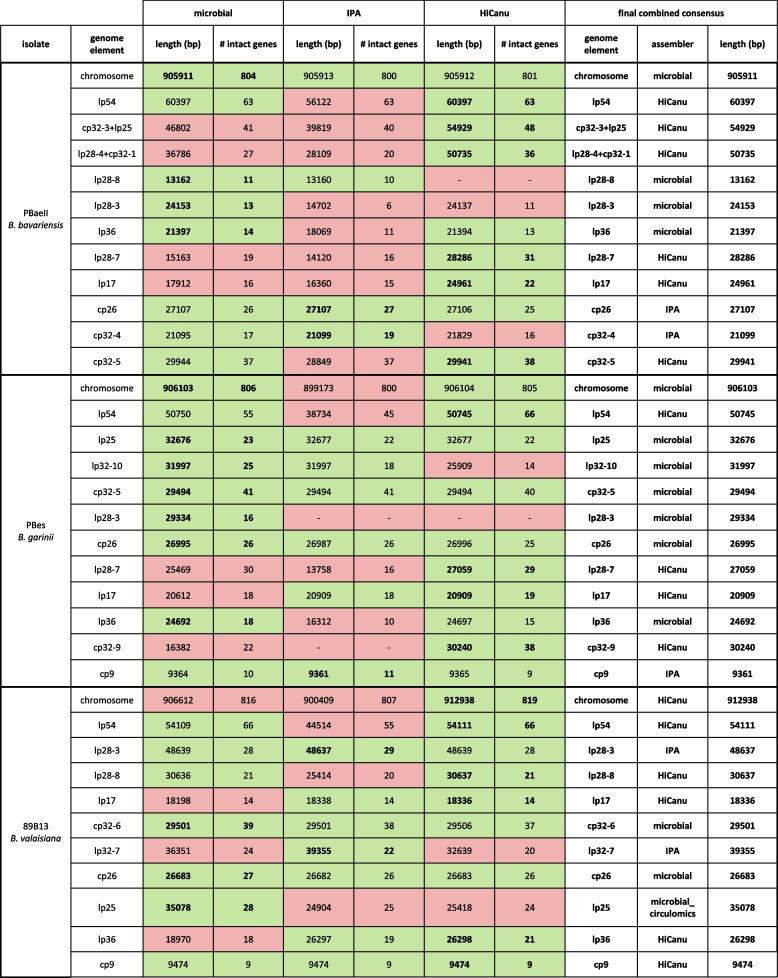
Assembly results after genome reconstruction of the three representative isolates (PBaeII, PBes and 89B13) for each assembler (microbial, IPA and HiCanu) and overview of the final combined consensus. Complete reconstructed genome elements are colored green, incomplete, missing or probably wrong assembled genome elements are shown in red. Genome elements used for final consensus are shown in bold

We conclude that *B. bavariensis* PBaeII contains the following genome elements (*n* = 12): a linear chromosome, 8 linear plasmids (lp54, cp32-3 + lp25, lp28-4 + cp32-1, lp28-8, lp28-3, lp36, lp28-7, lp17) and 3 circular plasmids (cp26, cp32-4, cp32-5) (Table 2, Fig. 4). The microbial assembler completely reconstructed 8 out of 12 genome elements, IPA only 4 and HiCanu reached a maximum of 9 complete genome elements (Table 3). The HiCanu assembly also contained a maximum of 1104 intact genes, followed by microbial and IPA with 1088 and 1064 intact genes, respectively (Table 3). The chromosome and the plasmid cp26 are the only genome elements that were successfully completely assembled by all three assemblers. Plasmids lp54, lp36 and cp32-5 were only completely reconstructed by the microbial and HiCanu assemblers and incomplete by the IPA assembler. Two plasmids (lp28-8 and cp32-4) were completely reconstructed by microbial and IPA assembler, while HiCanu failed to generate the complete cp32-4 and did not assemble the lp28-8 at all. The remaining five plasmids were only completed by one of the assemblers (HiCanu: cp32-3 + lp25, lp28-4 + cp32-1, lp28-7 and lp17; microbial: lp28-3).

**Table 3:** Assembler performance comparison with regard to the completeness of assembled genome elements

				**Microbial**			**IPA**			**HiCanu**		
Species	**Strain**	**# genome elements**	**# intact genes**	**# complete**	**# incomplete/** **missing**	**# intact genes**	**# complete**	**# incomplete/** **missing**	**# intact genes**	**# complete**	**# incomplete/** **missing**	**# intact genes**
*B. bavariensis*	**PBaeII**	**12**	**1126**	**8**	**4**	**1088**	**4**	**8**	**1064**	**9**	**3**	**1104**
*B. garinii*	**PBes**	**12**	**1118**	**9**	**3**	**1090**	**6**	**6**	**1007**	**10**	**2**	**1082**
*B. valaisiana*	**89B13**	**11**	**1095**	**7**	**4**	**1090**	**7**	**4**	**1064**	**9**	**2**	**1085**
total		**35**	**3339**	**24**	**11**	**3268**	**17**	**18**	**3135**	**28**	**7**	**3271**

*Borrelia garinii* PBes also has 12 genome elements: a linear chromosome, 7 linear plasmids (lp54, lp25, lp32-10, lp28-3, lp28-7, lp17, lp36) and 4 circular plasmids (cp26, cp32-5, cp32-9, cp9) (Table 2, Fig. 4). The plasmid lp32-10 carries the PFam32 locus of cp32-10 (circular plasmid), but has wraparounds at both ends (additional information, Figure S4) that include telomere sequences. The latter indicates a linear structure and the plasmid was therefore named lp32-10 instead of cp32-10. Plasmids that possess a cp32 type PFam32 locus, but have a linear structure have previously been described, including lp32-10 [17]. Out of 12 genome elements, the microbial, IPA and HiCanu assembler completely reconstructed 9, 6 and 10 genome elements, respectively (Table 3). The microbial and HiCanu assembly contained similar numbers of intact genes with 1090 and 1082, respectively, while the IPA consensus only showed 1007 intact genes (Table 3). The 4 plasmids lp25, cp32-5, cp26 and cp9 were fully assembled by all of the assemblers. Five genome elements were completed by two assemblers: Microbial and HiCanu completely assembled the chromosome, lp54 and lp36; microbial and IPA completed the lp32-10; IPA and HiCanu successfully assembled lp17. The remaining 3 plasmids were completely assembled only by one of the assemblers (microbial: lp28-3; HiCanu: lp28-7 and cp32-9), while the plasmid cp32-9 was completely missing in the IPA assembly. Interestingly, the lp28-3 was completely assembled by the microbial assembler but was missing from the IPA and HiCanu assemblies. Illumina read mapping confirmed the presence of plasmid lp28-3 but showed that the average coverage (9.6) was quite low in comparison to the other genome elements (average coverage ranging from 62.0 to 640.3).

*Borrelia valaisiana* 89B13 contains the following 11 genome elements: a linear chromosome, 7 linear plasmids (lp54, lp28-3, lp28-8, lp17, lp32-7, lp25, lp36) and 3 circular plasmids (cp26, cp32-6, cp9) (Table 2, Fig. 4). The microbial and IPA assembler completely assembled 7 of 11 genome elements, and HiCanu reached a maximum of 9 (Table 3). The microbial and HiCanu assemblies contained a similar high number of intact genes (1090 and 1085, respectively), while IPA assembly contained 1064 intact genes (Table 3). All of the assemblers successfully completed 4 plasmids (lp28-3, cp32-6, cp26 and cp9). Another 4 plasmids were completed by two assemblers: microbial and HiCanu fully assembled lp54 and lp28-8; IPA and HiCanu completed lp17 and lp36. The chromosome and the plasmids lp32-7 (dot plot of the linear plasmid containing a PFam32 locus of cp32-7: additional information, Figure S5) and lp25 were completely assembled only by one assembler (HiCanu, IPA and microbial, respectively). The lp25 was not completely assembled by sequences based on DNA extracted via the Maxwell method (see methods for details on DNA extraction), but a combination of the Circulomics Nanobind DNA extract and the microbial assembler led to a complete reconstruction of the plasmid. This plasmid contains a very long inverted terminal repeat with a central unique sequence, which may be challenging to assemble due to sequence similarity between the two halves of the inverted repeat (dot plot: additional information section, Figure S6).

In summary, none of the assemblers resulted in complete genome sequences for isolates PBaeII, PBes and 89B13. HiCanu generated the highest number of completely assembled genome elements resulting in 28 complete genome elements out of 35 in total (PBaeII *n* = 12, PBes *n* = 12, 89B13 *n* = 11; Table 3), followed by the microbial assembler resulting in 24 complete genome elements. The lowest number of fully reconstructed genome elements was assembled by the IPA assembler where only about half of the genome elements were fully assembled (17 complete, 18 incomplete). Similar results were observed regarding the number of intact assembled genes. HiCanu assemblies contained the highest number of intact genes 3271 out of 3339 in total (PBaeII *n* = 1126, PBes *n* = 11,118, 89B13 *n* = 1095; Table 3), followed by microbial and IPA (3268 and 3135 intact genes, respectively).

#### Generation of a final consensus by combining data

We manually compared the results of the individual genome elements of each genome with regard to completeness and correctness and combined the data to generate a final consensus for all genome elements. If more than one assembler successfully completely reconstructed the genome element, we used the genome element with the highest number of intact genes as we considered it to be most correct. For example, the chromosome of PBaeII was completely assembled by the microbial, IPA and HiCanu assembler with a length of 905,911 bp, 905,913 bp and 905,912 bp and 804, 800 and 801 intact genes, respectively (Table 2). All three assemblers assembled the chromosome completely, but as the microbial contig showed the highest number of intact genes, we used this contig in the final combined consensus. The plasmid cp32-4 of PBaeII was completely reconstructed by the microbial and IPA assembler, but the HiCanu contig had atypical terminal repeats (Table 2). We observed incomplete wraparounds remaining in the trimmed HiCanu contig (additional information, Figure S3 L) that were not present in the cp32-4 contigs assembled by the microbial and IPA assembler (additional information, Figure S1 M and Figure S2 M) and therefore considered this as misassembly. The misassembled terminal repeats led to an increase in contig length (PBaeII cp32-4 HiCanu: 21,829 bp) compared to the microbial and IPA contig (21,095 bp and 21,099 bp, respectively). Although the HiCanu assembler reconstructed cp32-4 with maximum contig length, the IPA contig contained the maximum number of intact genes (IPA: 19, microbial: 17, HiCanu: 16) and was used for the final consensus as we consider it to be more likely correct. The final combined consensus of PBaeII (12 genome elements) includes 6 genome elements assembled by the HiCanu assembler (lp54, cp32-3 + lp25, lp28-4 + cp32-1, lp28-7, lp17, cp32-5), 4 microbial contigs (chromosome, lp28-8, lp28-3, lp36) and 2 IPA contig (cp26, cp32-4) (Table 2).

By the same strategy, we generated final consensus genomes for PBes and 89B13. PBes (12 genome elements) is a combination of 7 microbial contigs, 4 HiCanu contigs and 1 IPA contig, and the final consensus of 89B13 (11 genome elements) consists of 6 HiCanu contigs, 3 microbial contigs (one of them is based on the Circulomics Nanobind DNA extract) and 2 IPA contigs. With regard to future genome comparison, the core genome (chromosome, lp54 and cp26) was reoriented as given in the type strain B31 of *B. burgdorferi* s.s.. To confirm that the genome elements were reconstructed and concatenated correctly, we mapped the PacBio HiFi reads on the final combined consensus and checked for equal coverage throughout the plasmid. The mapping graphs and mapping statistics of PBaeII are shown in the additional information (Table S3, S4 and Figure S7). Plasmid designations were confirmed by phylogenetic analyses based on PFam32 loci (alignment and phylogenetic tree are deposited in a repository; 10.6084/m9.figshare.23578785).

## Discussion

Generating a complete *Borrelia* genome assembly using a single assembly algorithm is challenging even with accurate long reads, such as PacBio HiFi data. This is in part due to the high number of, and sequence similarity between, plasmids that exist in some *Borrelia* species but also partially due to a changing plasmid repertoire and gene content on identical plasmids types within strains of the same species. Thus, even the presence of reference genomes – if available – does not prevent the need for genome de novo assembly [16–20, 29, 30].

For some bacterial species reports exist of complete genome assembly using only one sequencing and assembly strategy [53–55]. We need to verify this for *Borrelia* since previous studies had failed to achieve this [15, 29, 40, 46]. In this study, we used a high fidelity approach utilizing a variety of assembly tool(s), to improve the complete *Borrelia* genome reconstruction. Our aim was to generate finished, gap-free and high quality genomes including complete plasmids, as these are of critical importance for comparative genomic analyses [36, 37]. We therefore focused on the PacBio data and the recently introduced HiFi data.

As assemblers differ in their underlying algorithms, differences in output and suitability for organisms can be observed [37], we noticed that the used assemblers (microbial, IPA and HiCanu) showed differences in suitability for *Borrelia* genome reconstruction. The recognized differences included a trend toward low contig numbers in the IPA assemblies, a high contig number in HiCanu assemblies while the microbial assembler showed no trend regarding the generated contig number. A low contig number potentially indicates that each genome element is reconstructed as only one contig. However, in IPA assemblies this positive aspect was paired with high numbers of incomplete and missing plasmids. The high contig numbers in HiCanu assemblies was due to duplicates which may not pose a major problem for known and well investigated species but could be confounding in species whose genomes are not yet explored. At the same time, this assembler provided the highest number of complete genome elements and intact genes and duplicates could be deleted after their identification and confirmation. Although the HiCanu assembler is not specifically designed for plasmid reconstruction, we observed high suitability for completed *Borrelia* plasmid reconstruction after some refinement steps. The microbial assembler is designed for microbial plasmid reconstruction and includes a special plasmid workflow that may lead to the reconstruction of plasmids that may be missed by other assemblers. Our study showed that the microbial assembler performed about as well as the HiCanu assembler and that this assembler can reconstruct plasmids with low coverage that may be missed by other assemblers (including HiCanu) and therefore, this assembler is useful for *Borrelia* plasmid reconstruction. Regardless of the trends of suitability for *Borrelia* genome assembly, for the representative isolates PBaeII, PBes and 89B13 complete genomes could not be obtained using only one assembler. We therefore propose that for complete genome assembly of *Borrelia* a workflow is needed that includes all three assemblers and developed an ensemble pipeline that lead to finished and high-quality genomes (see Fig. 1). In the following, we give some examples that underline particularly well our proposal.

The first example is plasmid lp28-3 that is carried by all three isolates (PBaeII: 24,153 bp, PBes: 29,334 bp and 89B13: 48,639 bp). The lp28-3 of PBaeII was only completely assembled by the microbial assembler, while the IPA and HiCanu assemblers recovered an incomplete sequence. In PBes the microbial assembler was also the only assembler that successfully assembled the lp28-3 plasmid completely, IPA and HiCanu assemblers did not recover the plasmid sequence at all. If only IPA or HiCanu assemblies had been available, the existence of PBes plasmid lp28-3 would not have been discovered. The Illumina read mapping revealed a low coverage of the plasmid in PBes (average coverage of lp28-3: 9.61, average coverage of the other genome elements > 60). Due to the low coverage, the microbial assembler (based on PacBio subreads and includes an optimized workflow for plasmid assembly) was the only assembler that successfully reconstructed the plasmid and one could speculate that the plasmid was currently in the process of being lost and was not present in every single cell when DNA was isolated. Interestingly, in contrast to the problematic lp28-3 reconstruction in PBaeII and PBes, the lp28-3 of 89B13 was completely reconstructed by all three assemblers. This shows that the difficulty of assembling the same plasmid type in different strains can vary substantially.

Another challenging plasmid was the lp25 of 89B13, which contained a very long inverted terminal repeat with a central unique sequence. This type of inverted dimer structure has been reported previously, where the authors speculated that recombination or failed segregation after replication might be the origin for this complex structure [56]. In general, the Circulomics Nanobind DNA extraction method did not improve assembly results compared to the standard DNA extraction method (Maxwell). However, lp25 of 89B13 could only be completely recovered by the sequenced DNA extracted via the Circulomics Nanobind method in combination with the microbial assembler.

There are several examples where HiCanu was the only assembler successfully completely reconstruct genome elements. Four genome elements of PBaeII were only completely assembled by HiCanu (cp32-3 + lp25, lp28-4 + cp32-1, lp28-7 and lp17). Nevertheless, the assembler failed to correctly reconstruct three further plasmids (lp28-3, lp36, cp32-4). Although the IPA assembler’s suitability for *Borrelia* plasmid reconstruction seems to be limited, this assembler was the only one that completely reconstructed the lp32-7 of 89B13. This shows that even generally more suitable assemblers can fail to generate complete plasmid reconstruction and in some cases a less suitable assembler is the only successful one.

These observations illustrate that using only one assembler for *Borrelia* genome assembly is insufficient and several assemblers are needed to define a finished *Borrelia* genome that includes all plasmids. We therefore developed a workflow for *Borrelia* genome reconstruction that includes several sequencing data, assembling strategies and manual curation. This ensemble pipeline enables us to generate complete and reliable *Borrelia* reference sequences that can be used as a basis for comparative genome analyses. Furthermore, these reference sequences are suitable to optimize assembler parameters in further studies and verify new assembler tools.

## Conclusion

Despite the latest sequencing and assembly technologies, *Borrelia* genome assembly is still highly complex. Using only one assembler is insufficient and several refinement steps are needed to generate finished and reliable *Borrelia* genomes. We presented an ensemble pipeline that enables the complete reconstructions of *Borrelia* genomes and may be interesting for the assembly of other complex microbial genomes.

## Methods

### Strains

*Borrelia* isolates used for this study are from the isolate bank of the German National Reference Centre for *Borrelia* at the Bavarian Health and Food Safety Authority. The processed samples (*n* = 27) belong to the genospecies *B. bavariensis* (*n* = 16), *B. garinii* (*n* = 9) and *B. valaisiana* (*n* = 2). Results of one representative isolate per genospecies are shown. Initial quality checks are shown for a further 24 isolates to show that our findings are not limited to the three representative samples. Information of the origin of the three representative isolates is listed in Table 4; all samples are listed in Table S5 (additional information).

**Table 4:** Characteristics of representative *Borrelia* isolates for sequence assembly comparison. *I*. = *Ixodes*

Isolate	Species	Year of isolation	Passage	Country	Biological origin	Pathogenicity	Vector	Host
PBaeII	*B. bavariensis*	1990	11	Germany	human	pathogenic	*I. ricinus*	rodent
PBes	*B. garinii*	1989	8	Germany	human	pathogenic	*I. ricinus,* *I. persulcatus*	bird
89B13	*B. valaisiana*	2005	8	Germany	tick (*I. ricinus*)	none pathogenic	*I. ricinus*	bird

### Cultivation and DNA extraction

Strains were cultured in inhouse-made MKP medium using standard procedures [57]. Cultures were grown to a density of 1 × 10^8^ cells per mL and genomic DNA was extracted using the Maxwell® 16 LED DNA kit (Promega, Germany) according to the manufacturer’s protocol. Additionally, DNA was extracted from one isolate (89B13, *B. valaisiana*) with the Nanobind CBB Big DNA Kit (Circulomics, USA) and the Gram-negative bacteria—UHMW (ultrahigh molecular weight) protocol provided by the manufacturer to investigate whether the purification of longer DNA fragments improves the sequencing and genome assembly results (results not shown as method did not generally led to improvement). DNA quality (260/280 ratio of pure DNA is ~ 1.8) and double stranded DNA concentration were measured using a NanoDrop® 1000 photometer (Thermo Fisher Scientific, USA) and a Qubit® 3.0 fluorometer (Thermo Fisher Scientific, USA), respectively.

### Next generation sequencing

Every isolate was sequenced using two different technologies: The Pacific Biosciences (PacBio) single-molecule, real-time (SMRT) long-read technology and the Illumina short-read technology. The Circulomics DNA extract of 89B13 was only sequenced using PacBio long read technology.

Illumina sequencing was performed on an Illumina MiSeq platform (Illumina, USA). Libraries were prepared using the Nextera XT DNA Library Preparation Kit and the Nextera XT Index Kit (Illumina, USA) according to the manufacturer’s protocol. Library quality and quantity checks were performed on a Fragment Analyzer (Agilent, Germany) or an Agilent TapeStation (Agilent, Germany). After bead-based library normalization according to the manufacturer’s protocol, sequencing was carried out using a MiSeq Reagent Kit V2 (Illumina, USA) generating paired-end reads of 250 bp.

Pacific Biosciences sequencing was performed by the Norwegian Sequencing Center (www.sequencing.uio.no). Libraries of the Maxwell DNA extracts were prepared using Pacific Biosciences’ protocol for SMRTbell™ Libraries and PacBio® Barcoded Adapters for Multiplex SMRT® Sequencing. Genomic DNA was sheared to 12 kb fragments using g-tubes (Covaris), samples were pooled and final libraries were size selected (cutoff 3 kb) using Ampure PB beads. Libraries were sequenced on a Pacific Biosciences Sequel using Sequel Polymerase v3.0, SMRT cells v3 LR and Sequencing chemistry v3.0. Subreads were demultiplexed using the Demultiplex Barcodes pipeline on SMRT Link v7.0.0.63985 or v8.0.0.80529 (SMRT Link Analysis Services and GUI v7.0.0.63989 or v8.0.0.80502). Additionally, HiFi reads were generated using SMRT Tools v9.0.0.92188 software with minimum number of passes 3 and minimum accuracy 0.99. HiFi reads were demultiplexed using lima pipeline.

The library of the Circulomics DNA extract of 89B13 was prepared using Pacific Biosciences protocol for Multiplexed Microbial Libraries Using SMRTbell® ExpressTemplate Prep Kit 2.0. DNA was sheared to 10–16 kb fragments using g-tubes (Covaris) and the final library were size selected (cutoff 3 kb) using Ampure beads. The library was sequenced on a Pacific Biosciences Sequel II instrument using Sequel II Binding kit 2.0 and Sequencing chemistry v2.0. HiFi reads were generated using CCS pipeline (SMRT Link v10.2.0.133434) and default settings (minimum number of passes 3, minimum predicted accuracy 0.99). HiFi reads were demultiplexed using Demultiplex Barcodes pipeline on SMRT Link v10.2.0.133434.

### Genome assembly

Depending on the sequence data (PacBio subreads, PacBio HiFi reads, Illumina), different assembly methods were used for genome reconstruction as outlined below (workflow overview is shown in Fig. 1).

#### Microbial

PacBio subreads were assembled using the Microbial Assembly application with default settings on SMRT Link (v8.0.0.80529, SMRT Link Analysis Services and GUI v 8.0.0.80501, download: https://www.pacb.com/support/software-downloads/, user guide: https://www.pacb.com/wp-content/uploads/SMRT-Link-User-Guide-v8.0.pdf (page76)). The PacBio Microbial Assembly application was specifically developed for microbial genomes including plasmids. The pipeline reconstructs the chromosome in the first step, followed by a mapping and filtering of unmapped or poorly mapped reads which are then used for the downstream plasmid assembly. Due to this special plasmid workflow, the application may reconstruct plasmids that other assemblers may miss. To polish indels and sequencing errors that may be present in PacBio contigs, Illumina short reads were mapped to the PacBio microbial contigs and a consensus sequence was extracted using CLC Genomic Workbench v.21. The following settings were used for read mapping and consensus extraction: match score 1, mismatch cost 2, insertion and deletion cost 3, similarity fraction 0.8 and length fraction 0.5. If no Illumina reads mapped, the consensus sequence was filled from reference sequence (that is the PacBio microbial contig) and as conflict resolving strategy ‘vote‘ was used. For further contig polishing and to find plasmids that was presented in two contigs, an hybrid assembly of the short and long read data (Illumina reads and PacBio microbial contigs, respectively) was performed by hybridSPAdes (SPAdes v.3.14.1) [58, 59] using k-mer sizes of 21, 33, 55, 77, 99 and 127 (kmer sizes options are from Margos et al. (2017) and Becker et al. (2020) [15, 29]). The assembly is based on Illumina short-read assembly graph constructed by SPAdes, which hybridSPAdes uses in combination with long-read data (in our case the microbial contigs) to close gaps.

#### Improved Phase Assembler (IPA)

PacBio HiFi reads were assembled using the IPA HiFi genome assembler on SMRT Link Version 10.1.0.115488 with default settings (download: https://www.pacb.com/support/software-downloads/, github: https://github.com/PacificBiosciences/pbipa, wiki: https://github.com/PacificBiosciences/pbbioconda/wiki/Improved-Phased-Assembler, user guide: https://www.pacb.com/wp-content/uploads/SMRT_Link_User_Guide_v10.1.pdf). The assembler was designed to use the accuracy of PacBio HiFi reads to produce high quality assemblies of diploid organisms, but it also includes a haploid workflow. However, the assembler is a genome assembler that is not specifically designed for use on microbial genomes including numerous plasmids. Instead of polishing with Illumina reads, the assembly pipeline includes polishing steps based on HiFi reads using Racon v1.4.13 [60].

#### HiCanu

The PacBio HiFi reads were also assembled using the HiCanu assembler [61], Canu v2.1.1, and the options "genomeSize = 1.6 m -pacbio-hifi". The HiCanu assembler is a modification of the Canu assembler and was specifically developed for HiFi data (homopolymer compression, overlap-based error correction, and aggressive false overlap filtering), but not specifically for microbial genome reconstruction. As it is the case for IPA, HiCanu assemblies were polished with HiFi reads using Racon v1.4.20 [60] and was not combined with Illumina data.

### Assembly quality check (QC) and refinement steps

#### QUAST and Merqury

Assembly quality was evaluated using the quality assessment tool QUAST v5.0.2 with default setting [62]. Additionally, Merqury v1.3 (using k-mer = 15 for meryl database generation) [63] was used to investigate the completeness of the assemblies by comparing the HiFi reads of a sample with the assembled contig (the more read sequences are included in the assembly, the higher is the calculated completeness). As a first indication of the assembly quality, we used the number of assembled contigs. In an optimal case, the number of genome elements (previously sequenced *B. burgdorferi* s.l. isolates carry between 7 and 22 genome elements) correspond to the number of contigs, as each genome element is represented in one contig. However, since the number of genome elements may vary between isolates and species, this can only be a first guess. High quality assemblies (i) have contigs whose lengths correspond to the chromosome (approximately 0.9 Mb) and to the lengths of the plasmids, (ii) have a total length that matches the genome size (~ 1.5 Mb for *Borrelia*), (iii) have a high N50 value (sequence length of the shortest contig at 50% of the total assembly length), (iv) have a low L50 value (count of smallest number of contigs whose length sum makes up half of the genome size) and (v) have a high degree of completeness (all reads can be found in assembly contigs). On the other hand, low quality assemblies often show a high contig number and short contig length, resulting in low N50 and high L50 values.

#### Contig wraparound, telomere and terminal direct repeats analyses

*Borrelia* genomes contain linear as well as circular genome elements (linear chromosome, linear and circular plasmids). Linear genome elements (chromosome and linear plasmids) are terminated by covalently closed hairpin structures (telomeres including the TAGTATA motive) [26–28, 64, 65]. To determine the completeness of assembled genome elements, we checked for wraparound sequences (terminal inverted repeats) that indicate telomere sequences and for direct repeats at the contig ends. Wraparound sequences with a “properly” spaced TAGTATA consensus sequences 14 bp from the center of the wraparound inverted repeat (S. Casjens, to be published elsewhere) were considered to indicate telomeres of linear elements and terminal direct repeats were considered to indicate circularity. Wraparound and terminal direct repeats were identified by a web-based nucleotide NCBI-BLASTN with default parameters [51] analyses using the setting “align two or more sequences”. By aligning a contig with itself, similarities within the contig were searched resulting in a dot plot and alignment that provides visual and detailed nucleotide position information about wraparound and terminal direct repeats regions. Wraparounds and terminal direct repeats were trimmed for further analyses. To confirm removed sequences were indeed wraparounds/ terminal direct repeats and that the sequence were trimmed at the right positon, we realigned the removed sequences with the trimmed contig (MEGA6 using ClustalW and default settings) [66, 67]. Some assembled contig sequences of circular plasmids did not include such terminal direct repeat sequences and therefore could not be confirmed as complete. If terminal direct repeats were not present, the contig was extended with a polyN tail (5,000 bp) at both ends and PacBio long reads were mapped using CLC Genomic Workbench v.21. The following settings were used for read mapping: match score 1, mismatch cost 2, insertion and deletion cost 3, similarity fraction 0.8 and length fraction 0.5. The consensus sequence were extended by 1,000 bp at both sides, replacing the Ns with the mapped read sequence and as conflict resolving strategy ‘vote‘ was used. The extended consensus was then checked again for terminal direct repeat sequences, and, if present, confirmed the completeness and circularity of circular plasmids.

#### Plasmid typing

Plasmids are named according to their paralogous gene families (PFam) consisting of PFam32, PFam49, PFam50, PFam57/62 that refer to previously described gene families in *B. burgdorferi* s.s. B31 [17–20, 25]. We used BLAST v.2.2.31 [51, 68] and the algorithm BLASTN with default settings to identify PFam32 plasmid partition gene loci in the contigs. For initial analyses we used as queries the PFam32 gene sequences of *B. burgdorferi* s.s. strains B31, BOL26, JD1 and 118a [29]. When PFam32 loci were found and had at least 86.5% base pair identity and 90% hit coverage, we used the respective plasmid designation. If the PFam32 locus was not found in contigs of one assembly, the reasons for this may be: (1) The contig may be part of the chromosome (chromosomal PFam32 sequences were not included in the query), (2) the contig presents an plasmid that that was assembled in multiple contigs (PFam32 locus is presented on another contigs) and such plasmids can be completed by contig concatenation (see next section), (3) contig is a duplicate of a portion of another plasmid/contig where the PFam32 locus is not located, (4) some plasmids do not encode PFam32 (e.g., cp9 of some genospecies) but encode PFam49, 50 and/or 57/62, (5) the plasmid represents a previously undefined PFam32 type. If a plasmid does not have PFam32 locus it will be designated uncharacterized. In addition, some contigs showed multiple PFam32 loci indicating the presence of plasmid fusions (e.g. lp28-4 + cp32-1 and cp32-3 + lp25) [15, 29].

After finishing all QC and refinement steps and the final dataset had been compiled, plasmid designations were confirmed by phylogenetic analyses based on PFam32 loci. The sequences listed in Table 5 were used as references. The gene encoding the ParA family protein with locus tag A6J42_RS12750 from *Leptospira interrogans* serovar Copenhageni strain FDAARGOS 203 chromosome sequence (GenBank accession number CP020414.2) was used as outgroup to root the phylogenetic tree.

**Table 5:** Reference sequences for plasmid phylogenetic tree

Isolate	Genospecies	NCBI Biosample
20047	*B. garinii*	SAMN08918487
B31	*B. burgdorferi* s.s.	SAMN02603966
PBi	*B. bavariensis*	SAMN08918524
TPT2017	*B. turdi*	SAMN08919082 (QBLM01000001- QBLM01000013)
VS116	*B. valaisiana*	SAMN02436326

Briefly, this process includes the following steps (detailed explanation can be found in Kuleshov et al. (2020) [47]): (1) contig annotation using a local version of the NCBI Prokaryotic Genome Annotation Pipeline (PGAP, 2022–04-14.build6021) [52], (2) extraction of the protein coding and pseudogenes sequences, (3) running another annotation using a local version of InterProscan databases, (4) extraction of the PFam32, 49, 50 and 57/62 sequences, (5) extracted PFam32 nucleotide sequences (including pseudogenes) of the plasmids were submitted to a multiple sequence alignment using ClustalW 2.1 [69] (settings: cost matrix: IUB, gap open penalty: 15, gap extension penalty: 6.66), (6) inference of a maximum-likelihood tree and calculation of branch supports with the ultrafast bootstrap [70] implemented in the IQ-TREE v2.0.3 software [71] (GTR + F + R4 model for nucleotide substitution and 1000 bootstrap replicates).

The alignment and the phylogenetic tree are deposited in a repository (10.6084/m9.figshare.23578785). Plasmids with two sets of PFam32 loci are shown twice in the tree (e.g. 89B13 lp25, PBaeII lp28-4 + cp32-1, PBaeII cp32-3 + lp25, see also Fig. 4). The chromosome of reference strain TPT2017 had a right hand extension matching sequences of plasmid lp28-2 [43] and therefore its PFam32 clusters with plasmid lp28-2.

#### Duplicates, misassemblies and plasmids present in multiple contigs

The assemblers used here sometimes create “duplicate”, nearly identical contigs. To detect duplicates, every contig of each assembly was BLASTed against all the other contigs of the same assembly (NCBI-BLASTN with default settings) [51]. If the entire length of a contig matched to portions of another contig with a high identity (e.g. over 99%), it was assumed to be a duplicate. After confirmation by aligning the duplicate to the matching contig using an alignment tool (MEGA6 using ClustalW and default settings) [66, 67], the duplicate contig was deleted. Misassemblies were detect by atypical dot plots, sometimes missing PFam32 loci, uneven coverage or large gaps on an Illumina or HiFi read mapping and/or no matching Illumina reads at all. Additionally, if the supposedly misassembled contig was not present in the assemblies generated by the other two assemblers, it was deleted. Furthermore, the contigs of the same assembly were examined for overlapping sequences by BLAST analyses (NCBI-BLASTN with default settings) [51], which indicates that contigs belong to the same plasmid; contigs with overlaps were then concatenated to produce the final consensus. Moreover, the microbial assembler contigs were compared to the hybridSPAdes contigs (based on Illumina reads and microbial contigs) to identify low quality or incomplete microbial contigs that were then replaced by or concatenate with the hybridSPAdes contig.

#### Intact gene analyses

To calculate numbers of intact genes the NCBI annotator PGAP (2022–04-14.build6021) [52] was used. The calculations were performed for the three refined assemblies (microbial, IPA and HiCanu) per isolate and resulted in information about the number of intact genes of the individual genome elements.

#### Comparison of assembly results

Assembly results were compared based on the assembly quality and completeness results (QUAST and Merqury results, respectively; see “QUAST and Merqury” section above). Furthermore, the three refined assemblies (microbial, IPA and HiCanu) were manually compared with each other regarding presence, completeness and correctness of individual genome elements. Genome elements were considered as complete when telomeres and terminal direct repeats were found in linear and circular genome elements, respectively. Correctness were judged by comparing the individual genome elements to those generated by the others assemblers as well as the number of intact genes. The higher the number of intact genes found on a genome element, the more correctly it was considered.

#### Generating the final consensus

The final consensus was generated by combining results of the different assemblies, where the most complete and correct sequences were used. In the final consensus the core genome (chromosome, lp54 and cp26) were reoriented as reported for *B. burgdorferi* B31 to facilitate future genome comparisons. To confirm the correct reconstruction of the genome elements, PacBio HiFi reads were mapped on the final combined consensus using CLC genomic workbench v.21 with the following setting: match score 1, mismatch cost 2, insertion and deletion cost 3, similarity fraction 0.8 and length fraction 0.5. The output “reads track” and “stand-alone read mapping” were both generated. Based on the “reads track” output, mapping graphs were generated including read coverage graph tracks.

## Supplementary Information

**Additional file 1: Table S1.** QUAST and Merqury results. **Table S2.** Detailed information about assembly results after genome reconstruction of PBaeII, PBes and 89B13. **Table S3.** Summary of mapping statistics of PBaeII. **Table S4.** Mapping statistics for single genome elements of PBaeII. **Table S5.** List and characteristics of all isolates. **Figures S1-S3.** Dot plots *B. bavariensis* PBaeII. **Figure S4.** Dot plots *B. garinii* PBes, lp32-10, microbial assembler. **Figure S5.** Dot plot *B. valaisiana* 89B13, lp32-7, IPA. **Figure S6.** Dot plot *B. valaisiana* 89B13, lp25, microbial_circulomics. **Figure S7.** Mapping graphs of* B. bavariensis* PBaeII.

## Data Availability

The final consensus sequences of *B. bavariensis* PBaeII, *B. garinii* PBes and *B. valaisiana* 89B13 can be found in NCBI Genbank under the BioProject PRJNA327303. PBaeII: BioSample SAMN05328448, Accession CP117799-CP117810; PBes: BioSample SAMN05328475, Accession CP119347-CP119357; 89B13: BioSample SAMN05328504, Accession CP116874-CP116884.
