# Supplementary material for: A high fidelity approach to assembling the complex Borrelia genome
Source: BMC Genomics. 2023 Jul 17;24:401. doi: 10.1186/s12864-023-09500-4 (PMC10353223; doi:10.1186/s12864-023-09500-4)
Supplement: Supplementary file 1 — Additional file 1: Table S1. QUAST and Merqury results. Table S2. Detailed information about assembly results after genome reconstruction of PBaeII, PBes and 89B13. Table S3. Summary of mapping statistics of PBaeII. Table S4. Mapping statistics for single genome elements of PBaeII. Table S5. List and characteristics of all isolates. Figures S1-S3. Dot plots B. bavariensis PBaeII. Figure S4. Dot plots B. garinii PBes, lp32-10, microbial assembler. Figure S5. Dot plot B. valaisiana 89B13, lp32-7, IPA. Figure S6. Dot plot B. valaisiana 89B13, lp25, microbial_circulomics. Figure S7. Mapping graphs of B. bavariensis PBaeII. [file 12864_2023_9500_MOESM1_ESM.pdf]

Additional information

Hepner et al. 2023 ‘A high fidelity approach to assembling the complex *Borrelia* genome’

Tables

Table S1: QUAST and Merqury results

Table S2: Detailed information about assembly results after genome reconstruction of PBaeII, PBes and 89B13

Table S3: Summary of mapping statistics of PBaeII

Table S4: Mapping statistics for single genome elements of PBaeII

Table S5: List and characteristics of all isolates

Figures

Figures S1-S3: Dot plots *B. bavariensis* PBaeII

Figure S4: Dot plots *B. garinii* PBes, lp32-10, microbial assembler

Figure S5: Dot plot *B. valaisiana* 89B13, lp32-7, IPA

Figure S6: Dot plot *B. valaisiana* 89B13, lp25, microbial_circulomics

Figure S7: Mapping graphs of *B. bavariensis* PBaeII

QUAST and Merqury

Table S1: QUAST and Merqury results of the three assemblies (microbial. IPA and HiCanu) per isolate. Bbav = *B. bavariensis*, Bgar = *B. garinii*, Bval = *B. valaisiana*. Best, medium and worst values are colored green, yellow and red, respectively.

sample	species	# genome elements of final consensus	# contigs			Largest contig			Total length			N50			L50			completeness		
			microbial	IPA	HiCanu	microbial	IPA	HiCanu	microbial	IPA	HiCanu	microbial	IPA	HiCanu	microbial	IPA	HiCanu	microbial	IPA	HiCanu
A91S	Bbav	12	14	13	18	933662	891365	914788	1463609	1353737	1494932	933662	891365	914788	1	1	1	95.41	92.67	96.57
DK6	Bbav	11	14	12	19	911828	908961	928163	1435225	1351374	1453961	911828	908961	928163	1	1	1	97.07	94.92	97.92
Lubl25	Bbav	13	13	15	13	931806	548810	927006	1425581	1363126	1446087	931806	361596	927006	1	2	1	96.02	93.29	97.32
PBaeII	Bbav	12	14	13	21	885916	930217	930187	1431816	1284545	1560066	885916	930217	930187	1	1	1	94.40	92.15	96.80
PBar	Bbav	13	14	14	19	912815	910833	931839	1420949	1352203	1495753	912815	910833	931839	1	1	1	93.22	92.52	97.04
PBN	Bbav	12	15	12	14	935572	885996	931322	1601950	1314745	1510834	935572	885996	931322	1	1	1	98.16	93.94	98.20
PEi	Bbav	12	18	13	13	911479	936044	934087	1452898	1435755	1489941	911479	936044	934087	1	1	1	96.63	95.55	97.03
PHerl	Bbav	13	14	18	17	910178	632669	913376	1383047	1348223	1482966	910178	285627	913376	1	2	1	97.29	94.25	98.34
PLad	Bbav	13	17	11	21	913155	887202	907421	1574895	1431246	1563160	913155	887202	907421	1	1	1	97.25	93.59	97.54
PNeb	Bbav	13	14	15	20	932744	913352	916218	1419359	1336290	1492849	932744	913352	916218	1	1	1	93.74	91.31	95.20
PNi	Bbav	11	11	11	15	933146	887671	912477	1370408	1258672	1433636	933146	887671	912477	1	1	1	95.83	93.56	97.62
PRab	Bbav	12	15	12	17	894000	913617	929504	1447857	1324450	1437064	894000	913617	929504	1	1	1	98.34	96.28	98.64
PRof	Bbav	12	12	13	16	934437	912965	931489	1412922	1413764	1496201	934437	912965	931489	1	1	1	95.77	95.06	98.01
PTrob	Bbav	13	19	16	20	931004	815117	930025	1579661	1431237	1511535	931004	815117	930025	1	1	1	97.06	95.20	98.36
PWin	Bbav	12	16	12	13	899983	886619	927623	1439080	1281304	1465500	899983	886619	927623	1	1	1	93.97	90.13	94.92
PZwi	Bbav	12	12	12	15	910197	896066	916521	1403403	1392318	1487076	910197	896066	916521	1	1	1	95.31	94.07	96.79
PBes	Bgar	12	12	12	13	936464	913525	933687	1388680	1306556	1392049	936464	913525	933687	1	1	1	98.51	94.73	97.56
PBr	Bgar	12	16	15	18	930411	910356	915097	1586276	1492805	1490818	930411	910356	915097	1	1	1	95.82	93.25	95.71
PFr	Bgar	12	16	19	15	929898	624223	928265	1458009	1372139	1477210	929898	275769	928265	1	2	1	94.51	95.16	98.47
PHC	Bgar	13	14	13	16	888778	893656	931969	1364182	1350114	1462547	888778	893656	931969	1	1	1	96.79	95.12	98.18
PKi	Bgar	11	12	13	17	913240	472730	928021	1339211	1339493	1452335	913240	439251	928021	1	2	1	95.54	95.23	99.29
PLa	Bgar	13	13	14	17	932545	521691	913842	1444152	1379307	1486384	932545	413396	913842	1	2	1	97.66	94.16	96.62
PLi	Bgar	12	15	14	15	934155	890862	934468	1420269	1334918	1454160	934155	890862	934468	1	1	1	95.65	94.13	97.65
PMa	Bgar	8	11	11	10	914774	890549	932921	1248402	1139925	1288298	914774	890549	932921	1	1	1	99.20	96.22	99.71
PMe	Bgar	11	12	14	13	911024	909950	927768	1418059	1406365	1410370	911024	909950	927768	1	1	1	94.20	92.90	95.47
100B40	Bval	11	11	12	21	920949	922113	940003	1390353	1299601	1495260	920949	922113	940003	1	1	1	96.62	93.96	96.82
89B13	Bval	11	24	14	17	900887	900409	933876	1458247	1435972	1478764	900887	900409	933876	1	1	1	95.39	93.65	95.31

Generation of the final consensus.

Table S2: Detailed information about assembly results after genome reconstruction of the three representative isolates (PBaeII, PBes and 89B13) for each assembler (microbial, IPA and HiCanu) and overview of successful assembler and maximal numbers of intact genes and the final combined consensus. Complete reconstructed genome elements are colored green, incomplete, missing or probably wrong assembled genome elements are shown in red.

		microbial			IPA			HiCanu					final combined consensus		
**isolate**	**genome element**	**length (bp)**	**comment**	**# intact genes**	**length (bp)**	**comment**	**# intact genes**	**length (bp)**	**comment**	**# intact genes**	**complete plasmid**	**complete plasmid + maximal # intact genes**	**genome element**	**assembler**	**length (bp)**
PBaeII *B. bavariensis*	chromosome	905911	complete | concatenat	804	905913	complete	800	905912	complete	801	microbial / IPA / HiCanu	microbial	**chromosome**	**microbial**	**905911**
	lp54	60397	complete	63	56122	incomplete	63	60397	complete	63	microbial / HiCanu	microbial / HiCanu	**lp54**	**HiCanu**	**60397**
	cp32-3+lp25	46802	incomplete | concatenated with hybridSPAdes contig	41	39819	incomplete	40	54929	complete	48	HiCanu	HiCanu	**cp32-3+lp25**	**HiCanu**	**54929**
	lp28-4+cp32-1	36786	incomplete	27	28109	incomplete	20	50735	complete	36	HiCanu	HiCanu	**lp28-4+cp32-1**	**HiCanu**	**50735**
	lp28-8	13162	complete	11	13160	complete	10	-	missing	-	microbial / IPA	microbial	**lp28-8**	**microbial**	**13162**
	lp28-3	24153	complete	13	14702	incomplete	6	24137	too short? | concatenated	11	microbial	microbial	**lp28-3**	**microbial**	**24153**
	lp36	21397	complete	14	18069	incomplete	11	21394	complete	13	microbial / HiCanu	microbial	**lp36**	**microbial**	**21397**
	lp28-7	15163	incomplete	19	14120	incomplete	16	28286	complete	31	HiCanu	HiCanu	**lp28-7**	**HiCanu**	**28286**
	lp17	17912	incomplete	16	16360	incomplete	15	24961	complete | concatenated	22	HiCanu	HiCanu	**lp17**	**HiCanu**	**24961**
	cp26	27107	complete	26	27107	complete	27	27106	complete	25	microbial / IPA / HiCanu	IPA	**cp26**	**IPA**	**27107**
	cp32-4	21095	complete	17	21099	complete	19	21829	wrong wraparound?	16	microbial / IPA	IPA	**cp32-4**	**IPA**	**21099**
	cp32-5	29944	complete	37	28849	incomplete	37	29941	complete	38	microbial / HiCanu	HiCanu	**cp32-5**	**HiCanu**	**29941**
PBes *B. garinii*	chromosome	906103	complete	806	899173	incomplete	800	906104	complete	805	microbial / HiCanu	microbial	**chromosome**	**microbial**	**906103**
	lp54	50750	complete	55	38734	incomplete	45	50745	complete	66	microbial / HiCanu	HiCanu	**lp54**	**HiCanu**	**50745**
	lp25	32676	complete	23	32677	complete	22	32677	complete	22	microbial / IPA / HiCanu	microbial	**lp25**	**microbial**	**32676**
	lp32-10	31997	complete	25	31997	complete | concatenate	18	25909	incomplete	14	microbial / IPA	microbial	**lp32-10**	**microbial**	**31997**
	cp32-5	29494	complete	41	29494	complete	41	29494	complete	40	microbial / IPA / HiCanu	microbial / IPA	**cp32-5**	**microbial**	**29494**
	lp28-3	29334	complete | low coverage	16	-	missing	-	-	missing	-	microbial	microbial	**lp28-3**	**microbial**	**29334**
	cp26	26995	complete	26	26987	complete	26	26996	complete	25	microbial / IPA / HiCanu	microbial / IPA	**cp26**	**microbial**	**26995**
	lp28-7	25469	incomplete	30	13758	incomplete	16	27059	complete	29	HiCanu	HiCanu	**lp28-7**	**HiCanu**	**27059**
	lp17	20612	incomplete	18	20909	complete	18	20909	complete	19	IPA / HiCanu	HiCanu	**lp17**	**HiCanu**	**20909**
	lp36	24692	complete	18	16312	incomplete	10	24697	complete	15	microbial / HiCanu	microbial	**lp36**	**microbial**	**24692**
	cp32-9	16382	incomplete	22	-	missing	-	30240	complete	38	HiCanu	HiCanu	**cp32-9**	**HiCanu**	**30240**
	cp9	9364	complete	10	9361	complete	11	9365	complete	9	microbial / IPA / HiCanu	IPA	**cp9**	**IPA**	**9361**
89B13 *B. valaisiana*	chromosome	906612	incomplete | concatenate	816	900409	incomplete	807	912938	complete	819	HiCanu	HiCanu	**chromosome**	**HiCanu**	**912938**
	lp54	54109	complete | concatenate	66	44514	incomplete	55	54111	complete	66	microbial / HiCanu	microbial / HiCanu	**lp54**	**HiCanu**	**54111**
	lp28-3	48639	complete	28	48637	complete	29	48639	complete	28	microbial / IPA / HiCanu	IPA	**lp28-3**	**IPA**	**48637**
	lp28-8	30636	complete	21	25414	incomplete	20	30637	complete	21	microbial / HiCanu	microbial / HiCanu	**lp28-8**	**HiCanu**	**30637**
	lp17	18198	incomplete	14	18338	complete	14	18336	complete	14	IPA / HiCanu	IPA / HiCanu	**lp17**	**HiCanu**	**18336**
	cp32-6	29501	complete	39	29501	complete	38	29506	complete	37	microbial / IPA / HiCanu	microbial	**cp32-6**	**microbial**	**29501**
	lp32-7	36351	incomplete	24	39355	complete	22	32639	incomplete	20	IPA	IPA	**lp32-7**	**IPA**	**39355**
	cp26	26683	complete	27	26682	complete	26	26683	complete	26	microbial / IPA / HiCanu	microbial	**cp26**	**microbial**	**26683**
	lp25	35078	complete | replaced by microbial_circulomics	28	24904	incomplete	25	25418	incomplete	24	microbial_circulomics	microbial_circulomics	**lp25**	**microbial_circulomics**	**35078**
	lp36	18970	incomplete	18	26297	complete | concatenate	19	26298	complete	21	IPA / HiCanu	HiCanu	**lp36**	**HiCanu**	**26298**
	cp9	9474	complete	9	9474	complete	9	9474	complete	9	microbial / IPA / HiCanu	microbial / IPA / HiCanu	**cp9**	**HiCanu**	**9474**

Mapping statistics of* B. bavariensis *PBaeII

Table S3: Summary of mapping statistics of PBaeII

	Count	Percentage of reads (%)	Average length	Number of bases	Percentage of bases (%)
References	12	-	105173.25	1262079	-
Mapped reads	8903	99.03	9131.71	82919924	98.49
Not mapped reads	87	0.97	14569.34	1267533	1.51
Total reads	8990	100.00	9364.57	84187457	100.00

Table S4: Mapping statistics for single genome elements of PBaeII

Genome element	Consensus length	Total read count	Average coverage
chromosome	905912	3926	28.65
lp54	60397	823	98.41
cp32-3+lp25	54929	533	71.09
lp28-4+cp32-1	50735	436	59.33
lp28-8	13162	78	34.73
lp28-3	24153	354	100.02
lp36	21397	275	91.28
lp28-7	28286	392	95.95
lp17	24961	1931	533.65
cp26	27107	72	13.61
cp32-4	21099	42	8.82
cp32-5	29941	41	7.54

Borrelia *isolates*

Table S5: List and characteristics of all isolates. *I.* = *Ixodes*

Sample	Species	Year of isolation	Passage	Country	Biological origin
A91S	*B. bavariensis*	1996	14	Netherlands	human
DK6	*B. bavariensis*	1990	12	Denmark	human
Lubl25	*B. bavariensis*	1995	16	Slovenia	human
PBaeII	*B. bavariensis*	1990	11	Germany	human
PBar	*B. bavariensis*	1988	8	Germany	human
PBN	*B. bavariensis*	1999	9	Germany	human
PEi	*B. bavariensis*	1994	21	Germany	human
PHerI	*B. bavariensis*	1989	10	Germany	human
PLad	*B. bavariensis*	2000	4	Germany	human
PNeb	*B. bavariensis*	1988	9	Germany	human
PNi	*B. bavariensis*	2000	7	Germany	human
PRab	*B. bavariensis*	1994	15	Austria	human
PRof	*B. bavariensis*	1989	2	Germany	human
PTrob	*B. bavariensis*	1988	17	Slovenia	human
PWin	*B. bavariensis*	1987	12	Germany	human
PZwi	*B. bavariensis*	1994	14	Germany	human
PBes	*B. garinii*	1989	8	Germany	human
PBr	*B. garinii*	1985	7	Germany	human
PFr	*B. garinii*	1995	6	Germany	human
PHC	*B. garinii*	1996	11	Germany	human
PKi	*B. garinii*	1992	16	Germany	human
PLa	*B. garinii*	1988	12	Germany	human
PLi	*B. garinii*	1988	15	Germany	human
PMa	*B. garinii*	1989	9	Jugoslavia	human
PMe	*B. garinii*	1988	5	Germany	human
100B40	*B. valaisiana*	2005	6	Germany	tick (*I. ricinus*)
89B13	*B. valaisiana*	2005	8	Germany	tick (*I. ricinus*)

Figures S1-S3: *Dot plots* B. bavariensis *PBaeII*

Figure S1: microbial assembler. Panels A-N: individual contigs as indicated.

Figure S2: IPA assembler. Panels A-M: individual contigs as indicated.

Figure S3: HiCanu assembler. Panels A-U: individual contigs as indicated.

Dot plots of the single contigs of the microbial, IPA and HiCanu assembler are shown untrimmed (left) and trimmed (right). In the case that no wraparounds or direct terminal repeats are present the untrimmed contig, both dot plots are the same.

Figure S1: microbial assembler

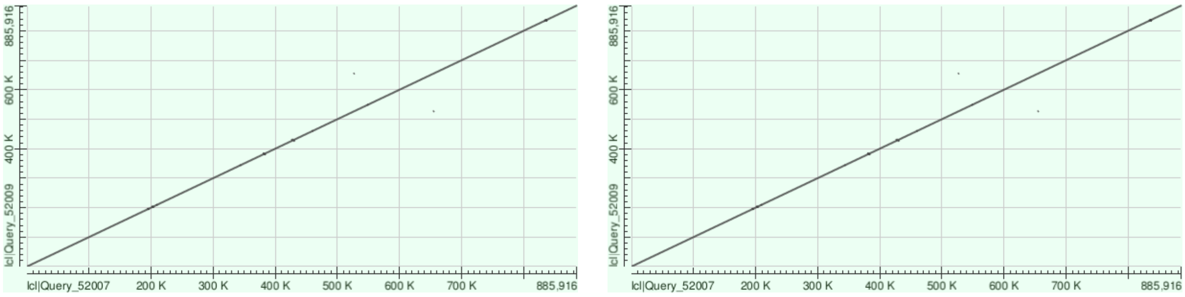

Figure S1 A: dot plots ctg.s1.000000F (part of chromosome)

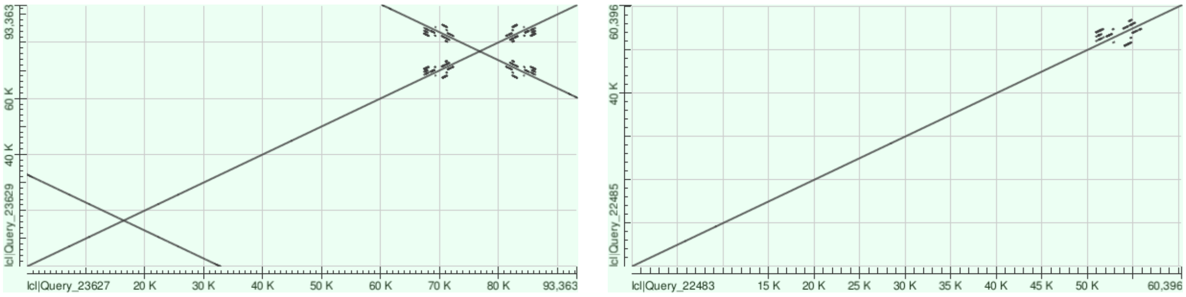

Figure S1 B: ctg.s2.000000F (lp54)

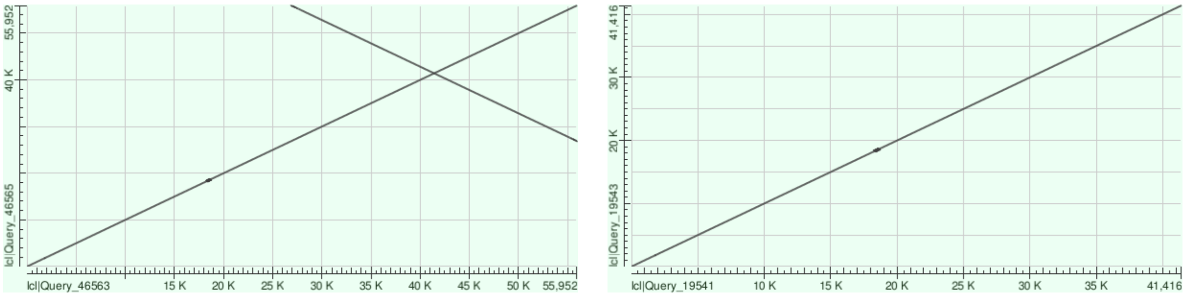

Figure S1 C: ctg.s2.000001F (part of cp32-3+lp25_incomplete)

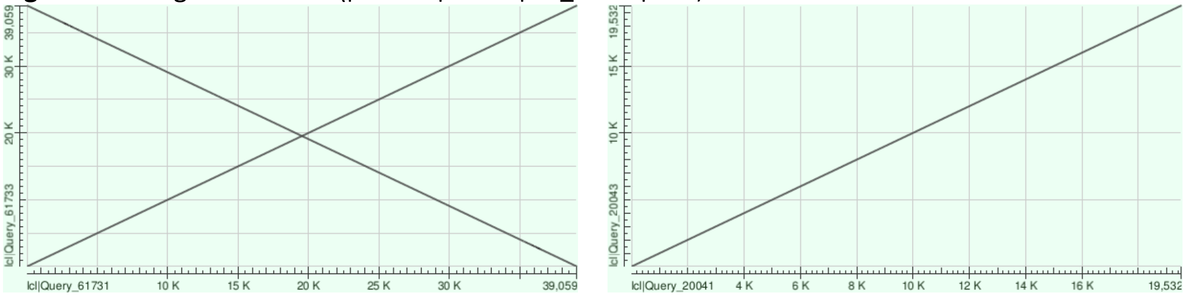

Figure S1 D: ctg.s2.000002F (part of chromosome)

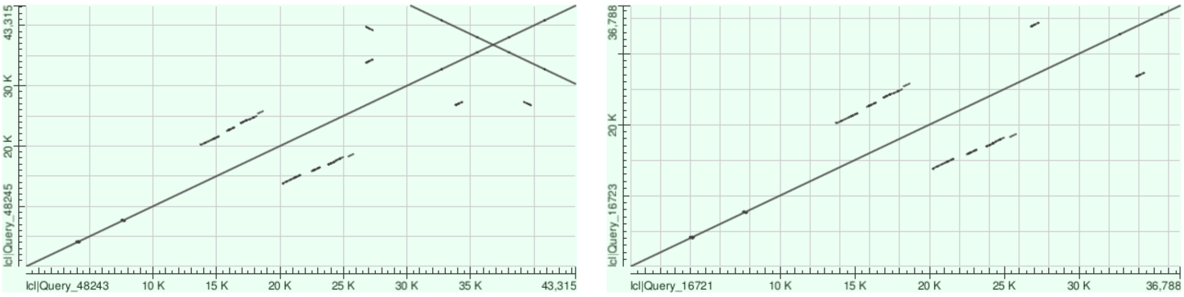

Figure S1 E: ctg.s2.000003F (lp28-4+cp32-1 _incomplete)

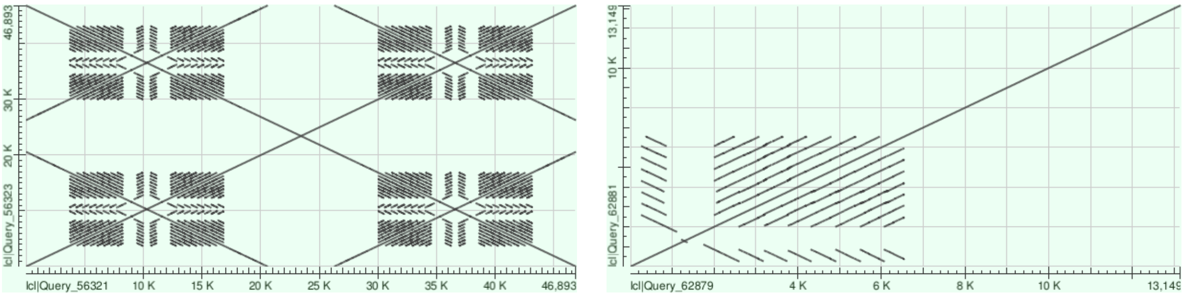

Figure S1 F: ctg.s2.000004F (lp28-8)

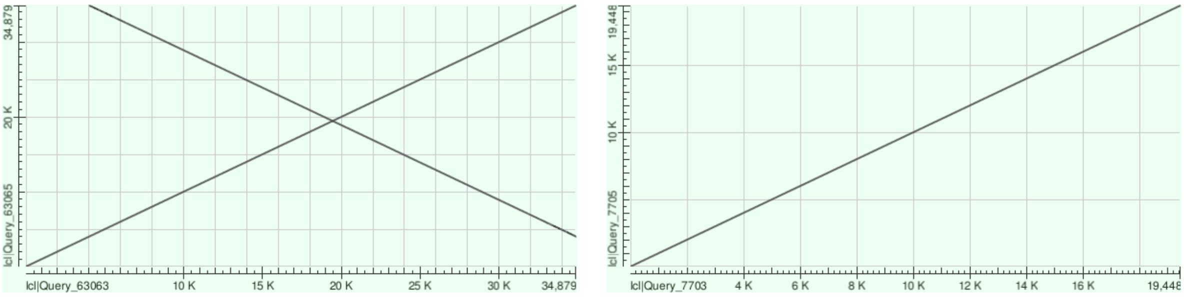

Figure S1 G: ctg.s2.000005F (part of chromosome)

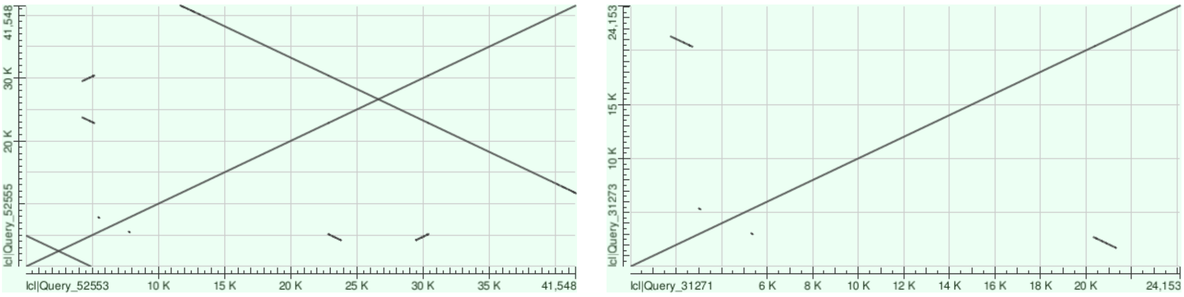

Figure S1 H: ctg.s2.000006F (lp28-3)

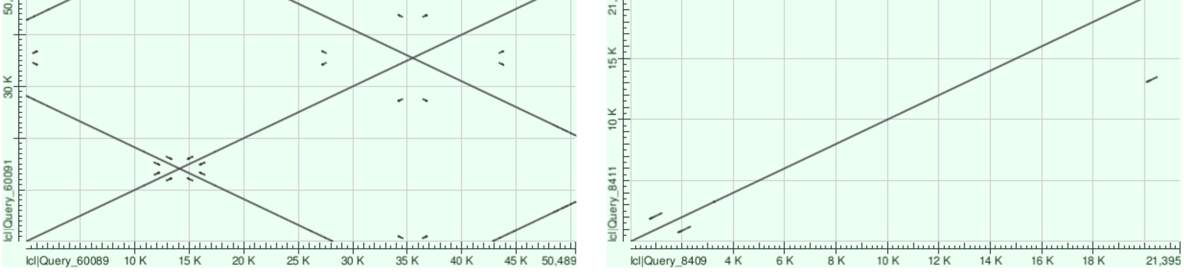

Figure S1 I: ctg.s2.000007F (lp36)

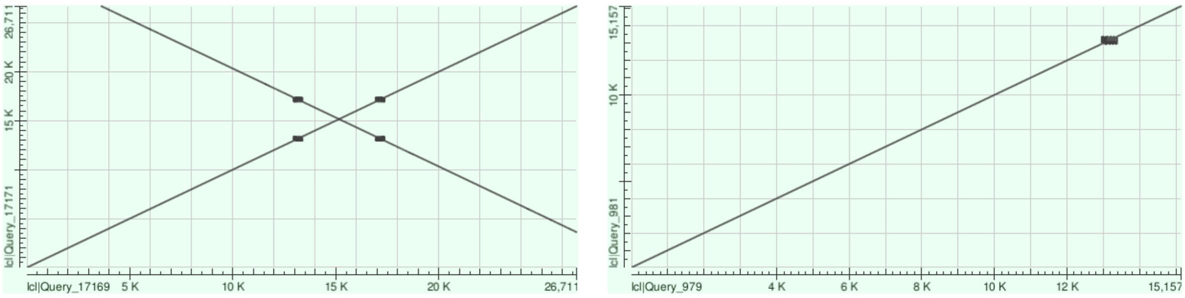

Figure S1 J: ctg.s2.000008F (lp28-7_incomplete)

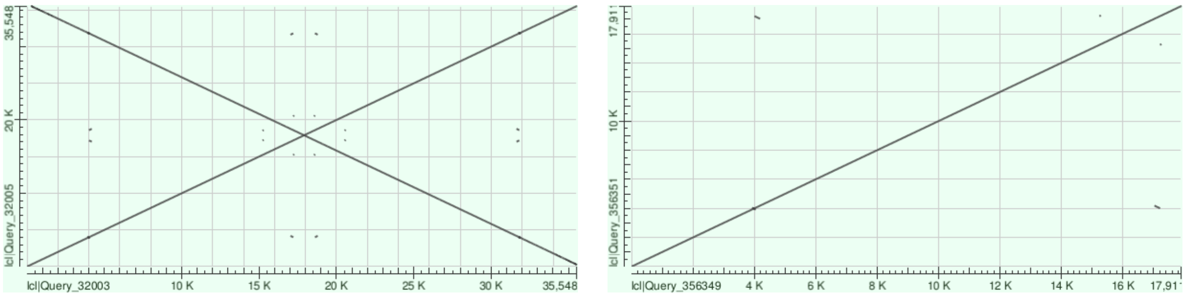

Figure S1 K: ctg.s2.000009F (lp17_incomplete)

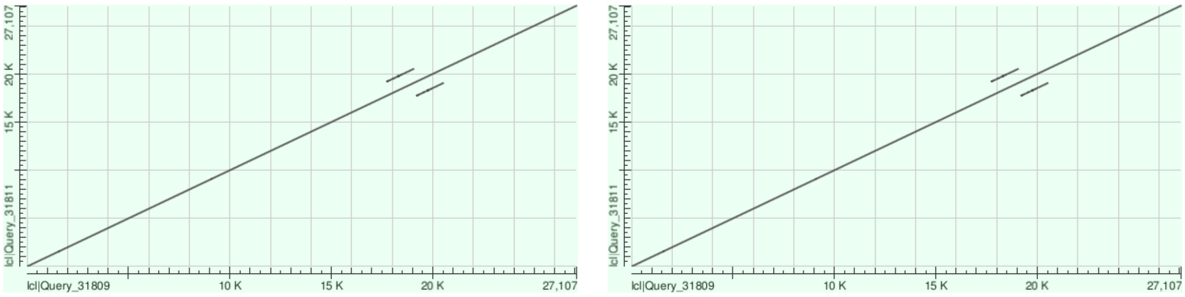

Figure S1 L: ctg.s2.10arro (cp26)

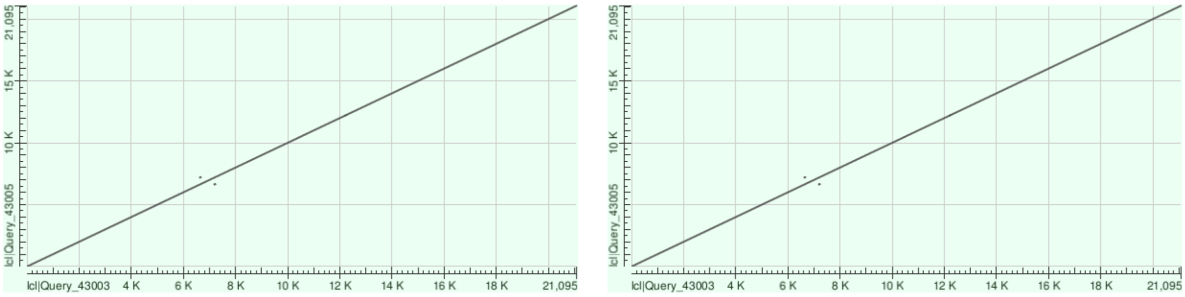

Figure S1 M: ctg.s2.12arro (cp32-4)

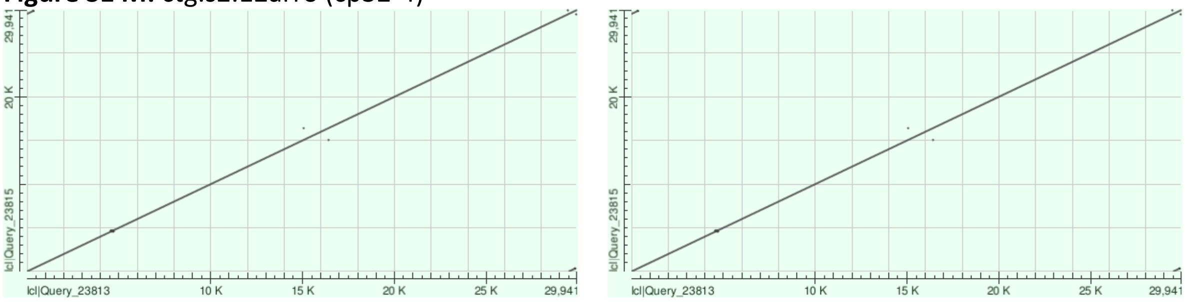

Figure S1 N: ctg.s2.14arro (cp32-5)

Figure S2: IPA assembler

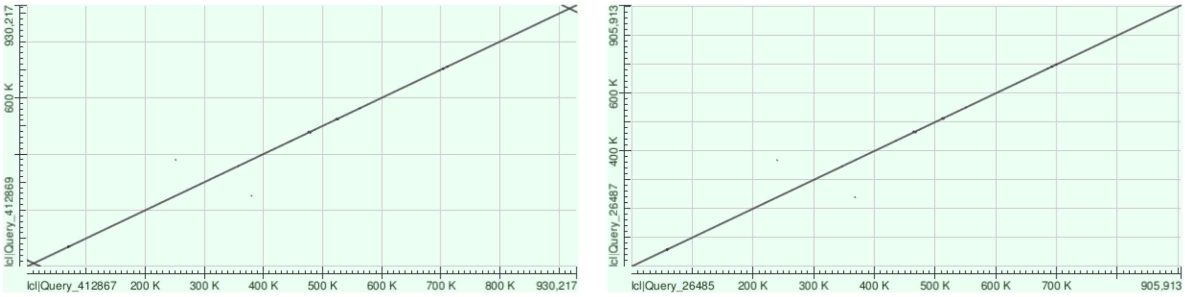

Figure S2 A: ctg.000000F (chromosome)

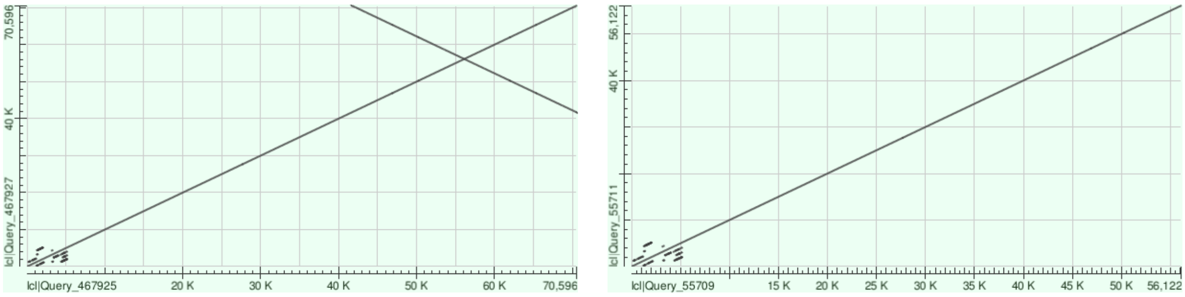

Figure S2 B: ctg.000001F (lp54_incomplete)

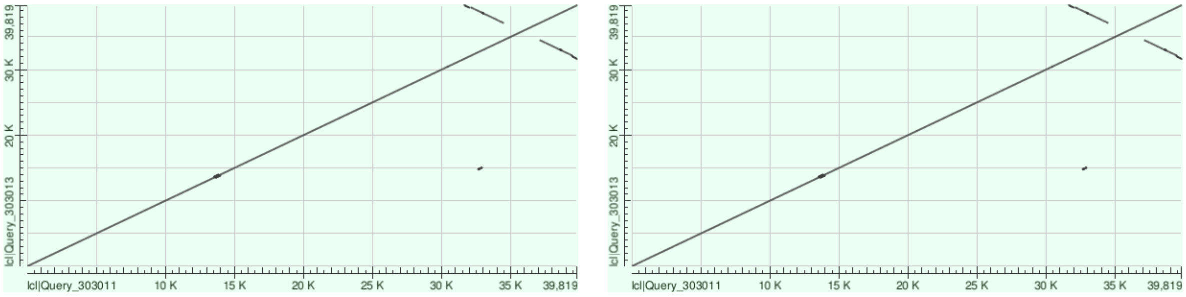

Figure S2 C: ctg.000002F (cp32-3+lp25_incomplete)

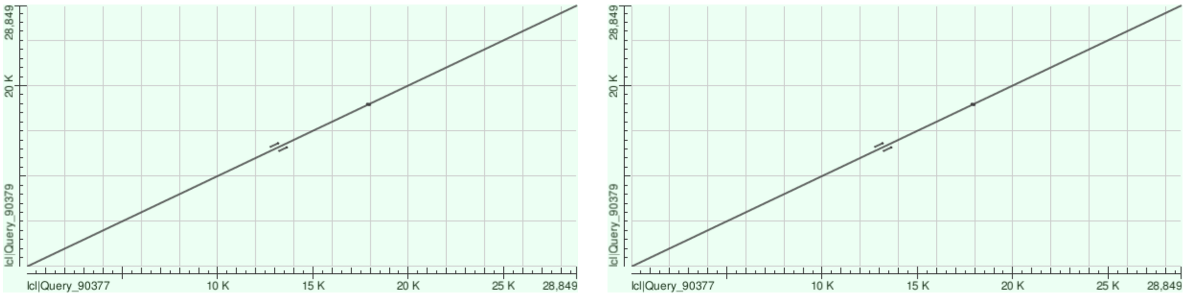

Figure S2 D: ctg.000003F (cp32-5)

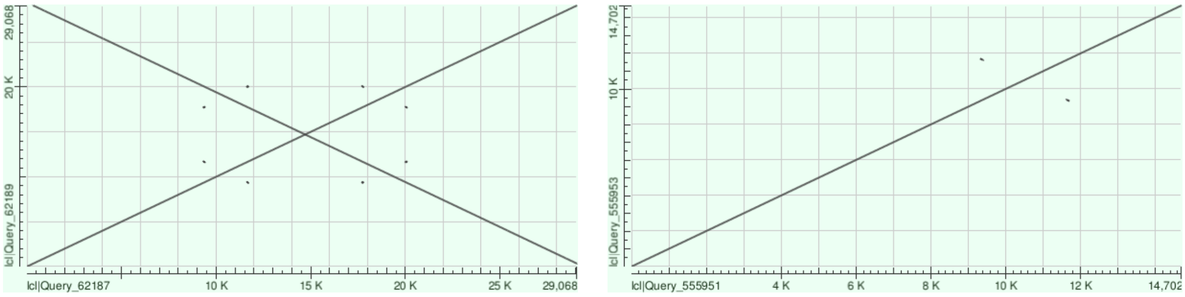

Figure S2 E: ctg.000004F (lp28-3_incomplete)

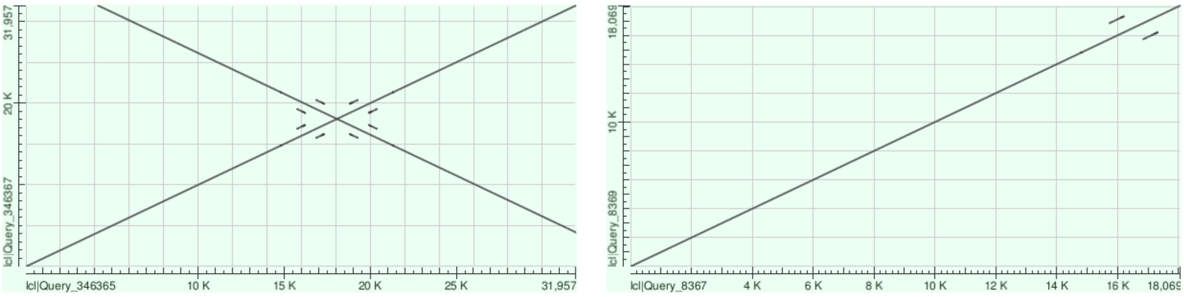

Figure S2 F: ctg.000005F (lp36_incomplete)

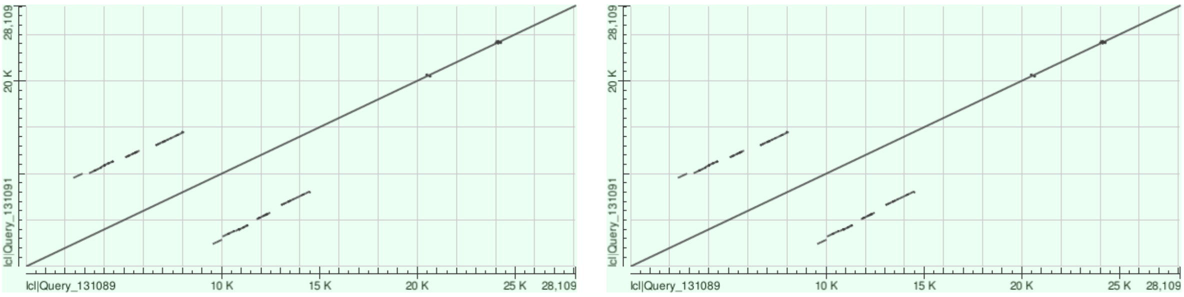

Figure S2 G: ctg.000006F (lp28-4+cp32-1 _incomplete)

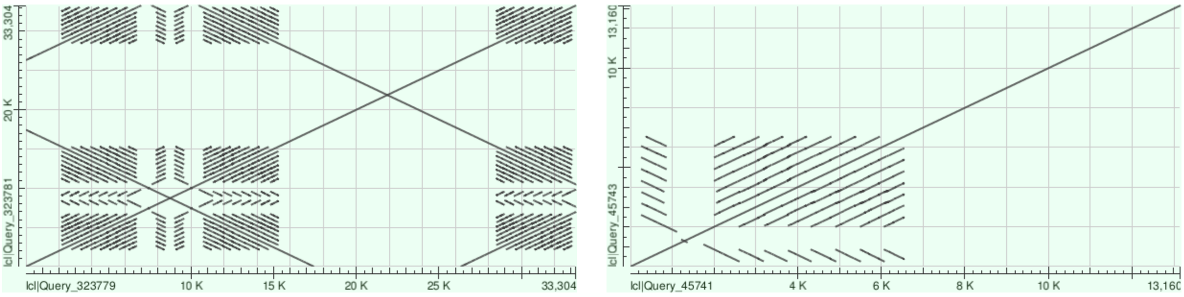

Figure S2 H: ctg.000007F (lp28-8)

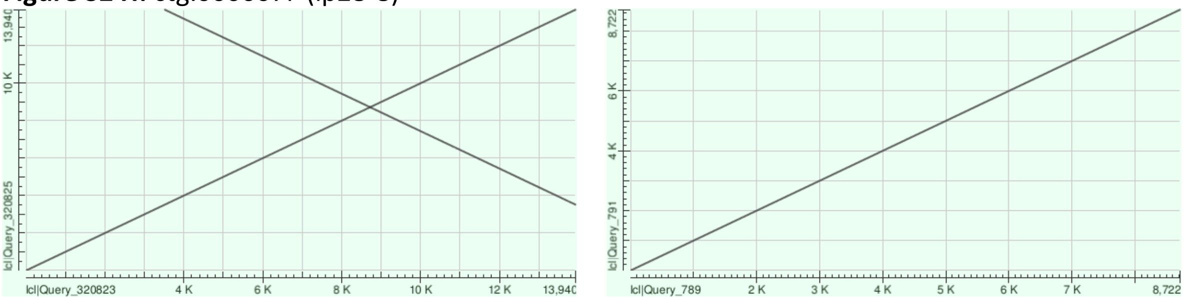

Figure S2 I: ctg.000008F (duplicate to lp28-4+cp32-1 _incomplete)

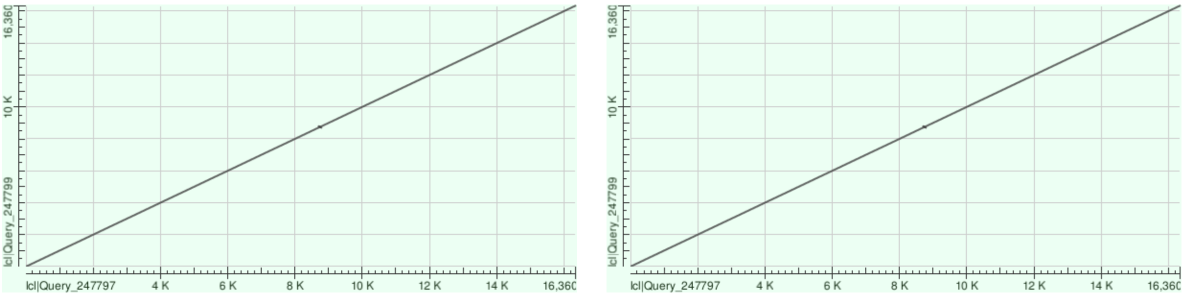

Figure S2 J: ctg.000009F (lp17_incomplete)

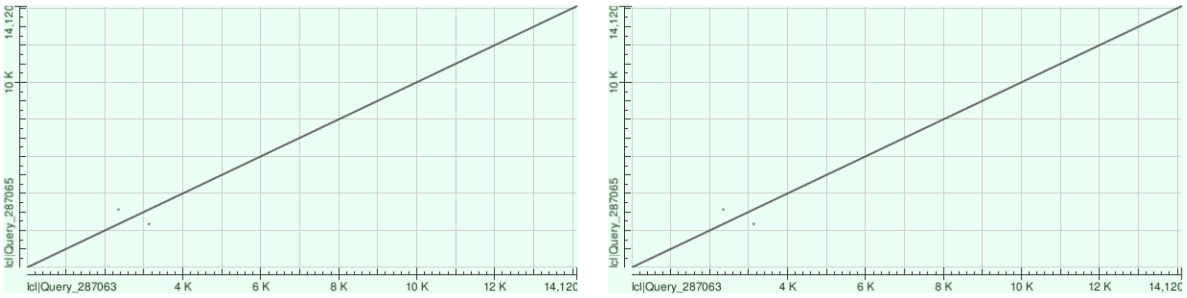

Figure S2 K: ctg.000010F (lp28-7_incomplete)

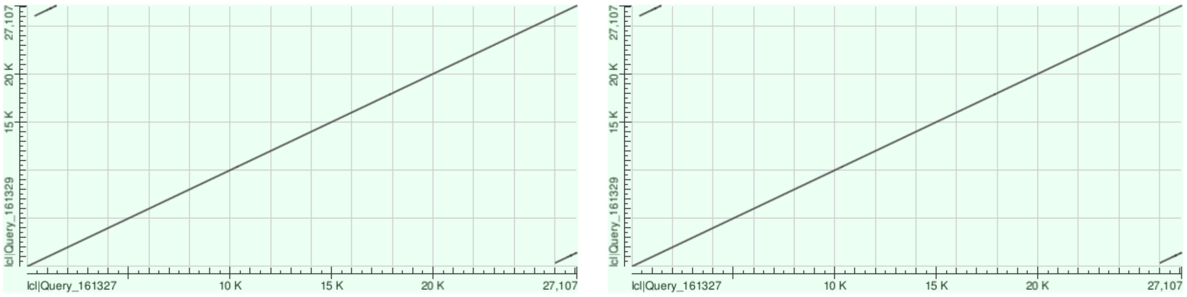

Figure S2 L: ctg.11 (cp26)

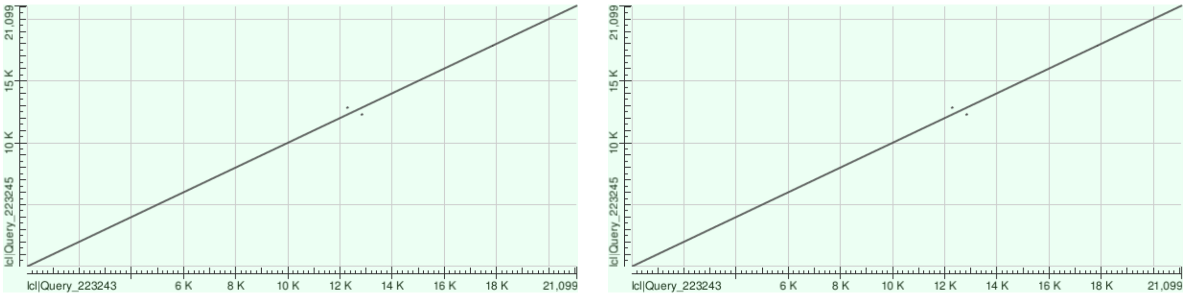

Figure S2 M: ctg.13 (cp32-4)

Figure S3: HiCanu assembler

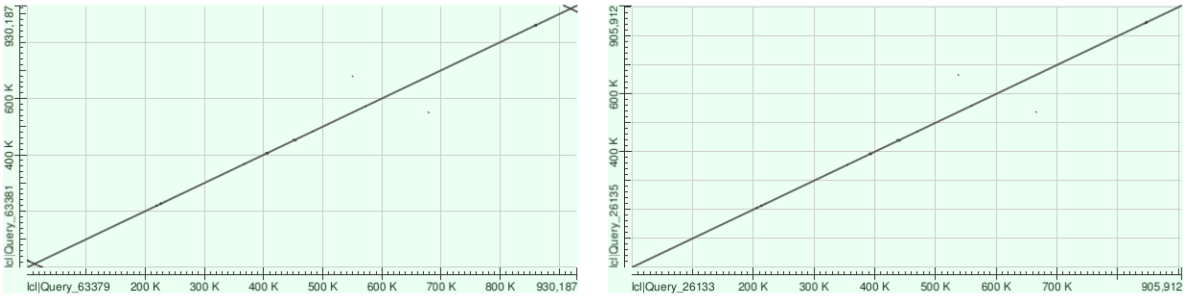

Figure S3 A: tig00000001 (chromosome)

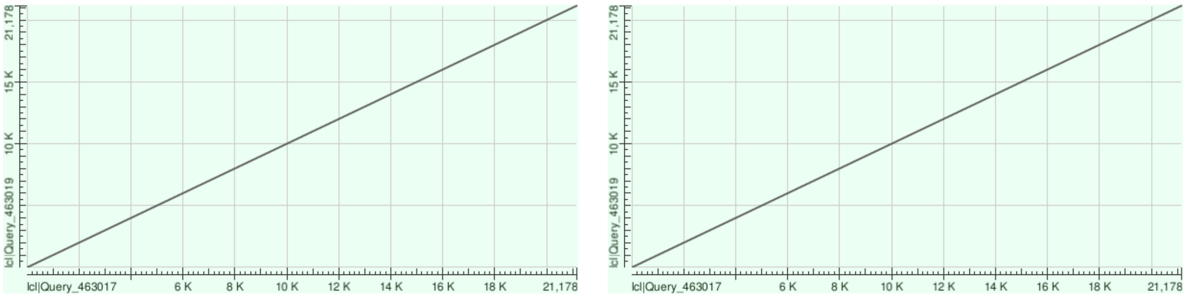

Figure S3 B: tig00000003 (duplicate to chromosome)

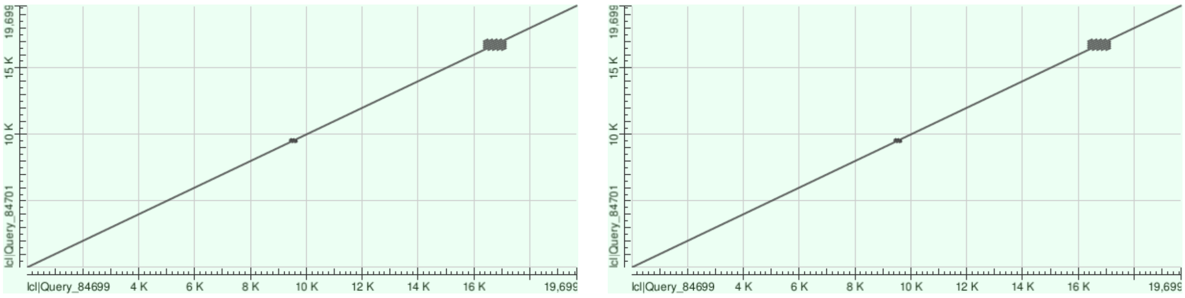

Figure S3 C: tig00000004 (duplicate to chromosome)

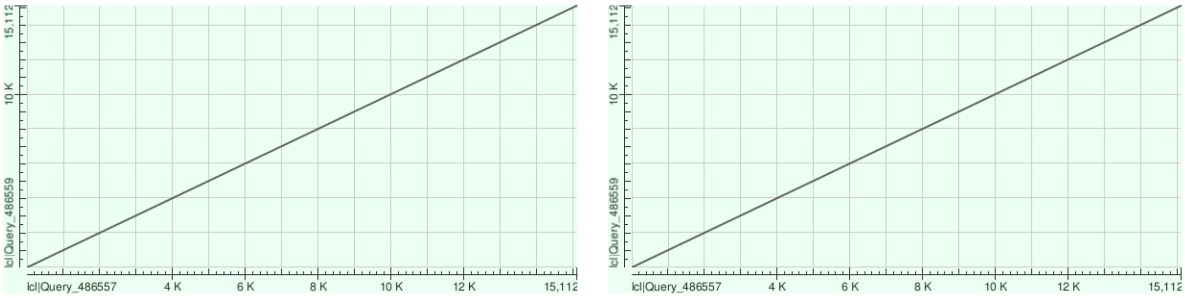

Figure S3 D: tig00000005 (duplicate to chromosome)

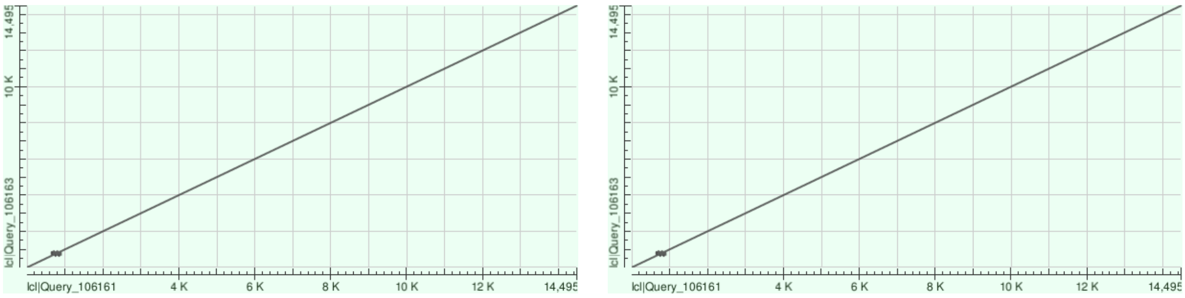

Figure S3 E: tig00000006 (duplicate to chromosome)

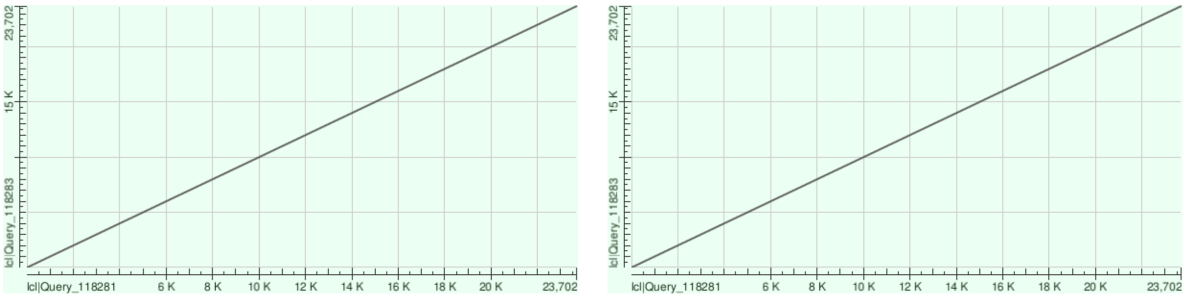

Figure S3 F: tig00000008 (duplicate to chromosome)

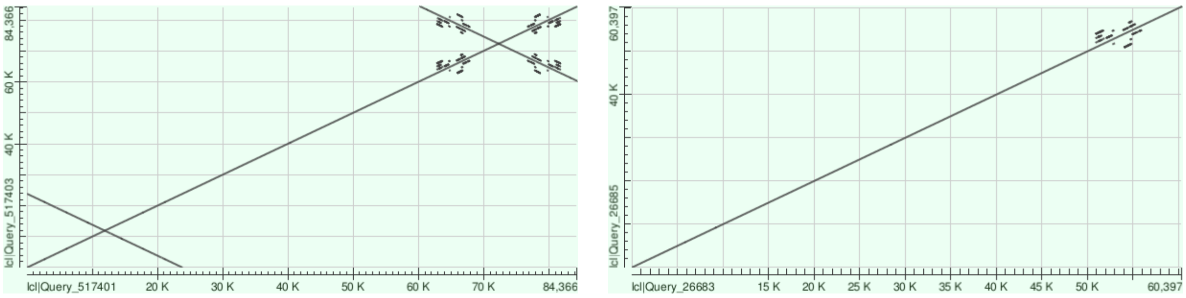

Figure S3 G: tig00000009 (lp54)

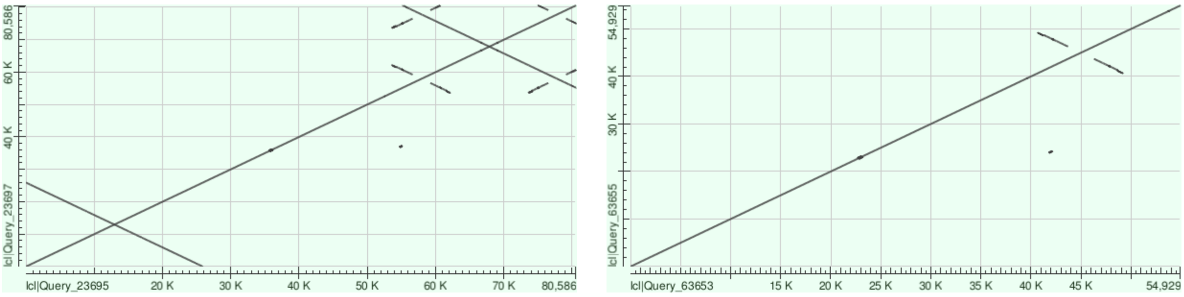

Figure S3 H: tig00000010 (cp32-3+lp25)

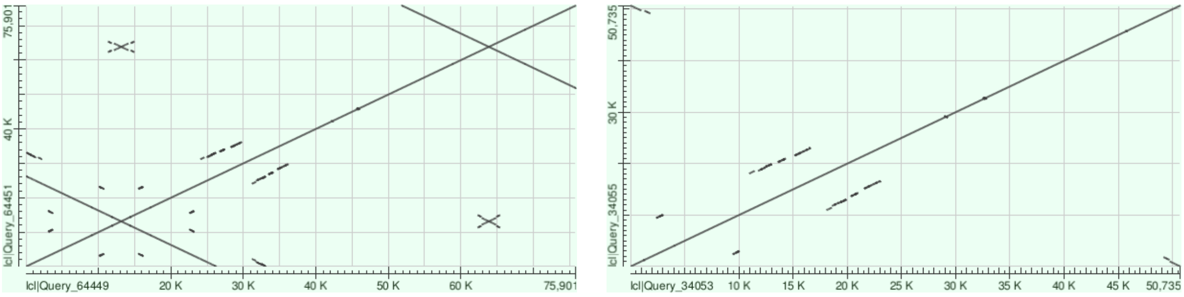

Figure S3 I: tig00000011 (lp28-4+cp32-1)

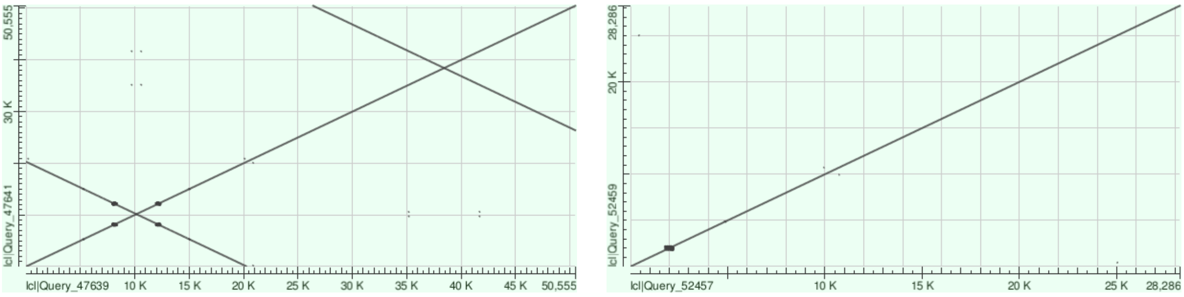

Figure S3 J: tig00000012 (lp28-7)

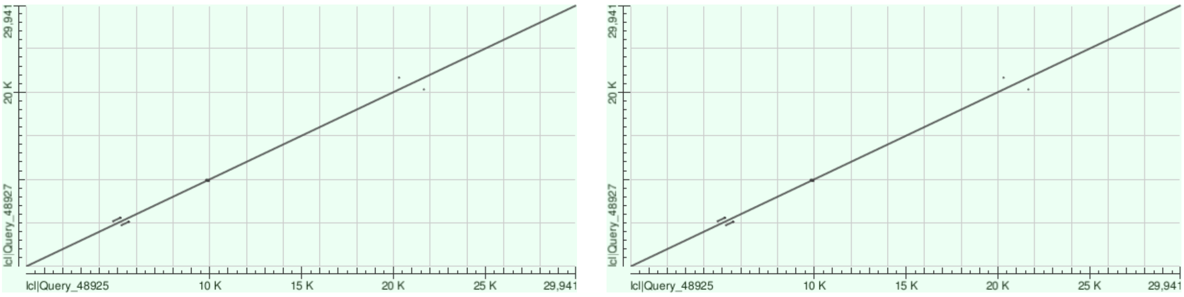

Figure S3 K: tig00000014 (cp32-5)

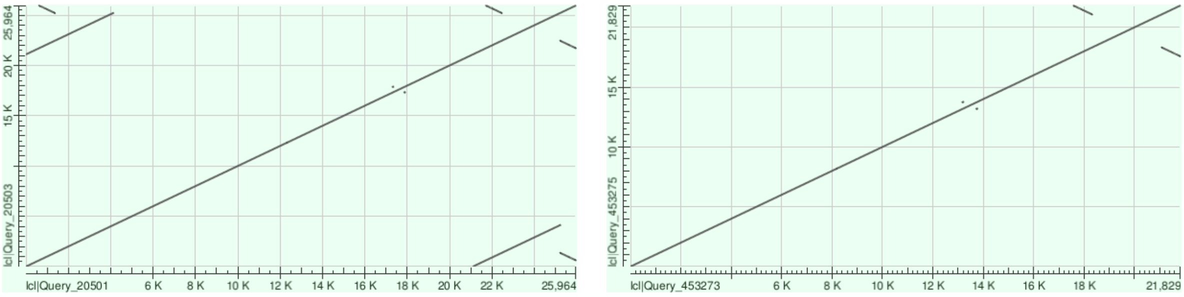

Figure S3 L: tig00000015 (cp32-4)

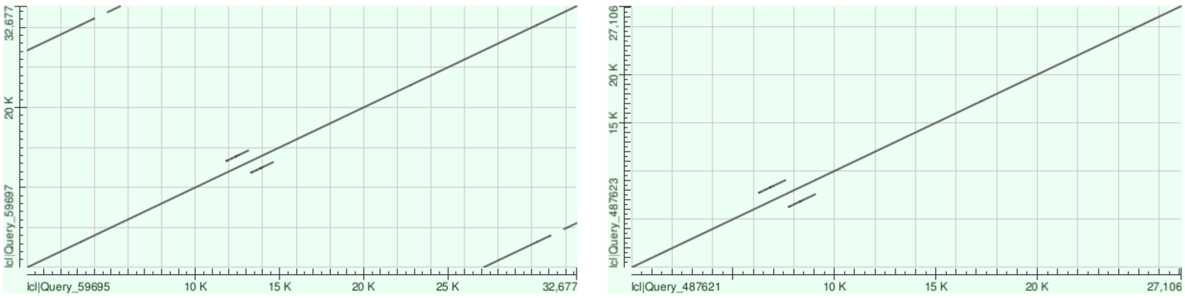

Figure S3 M: tig00000016 (cp26)

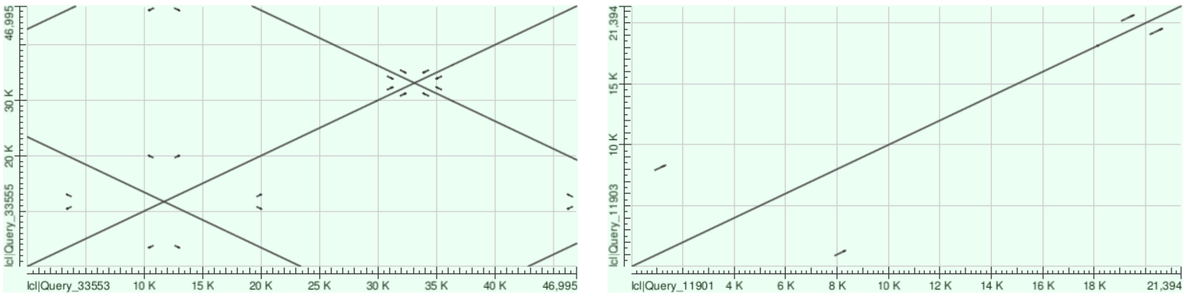

Figure S3 N: tig00000018 (lp36)

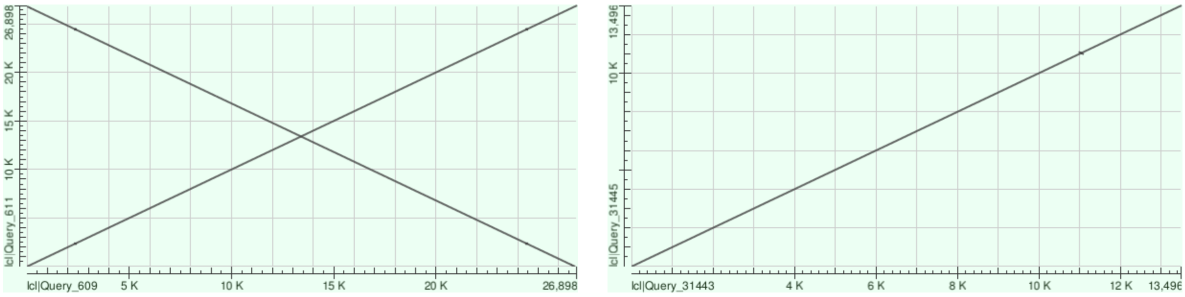

Figure S3 O: tig00000019 (part of lp17)

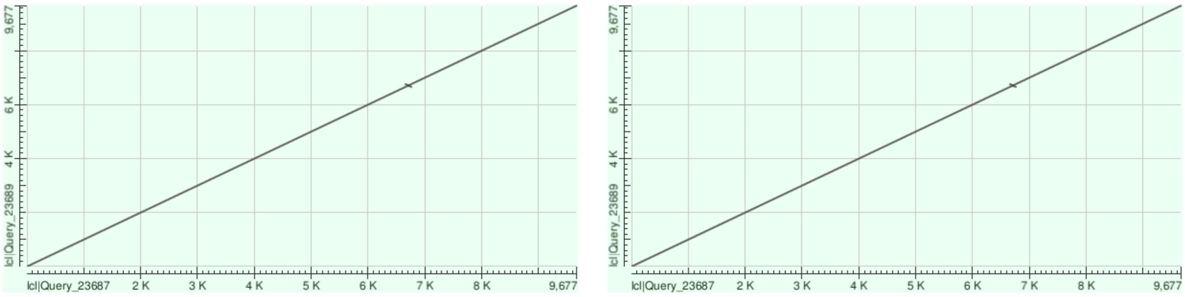

Figure S3 P: tig00000020 (duplicate to lp17)

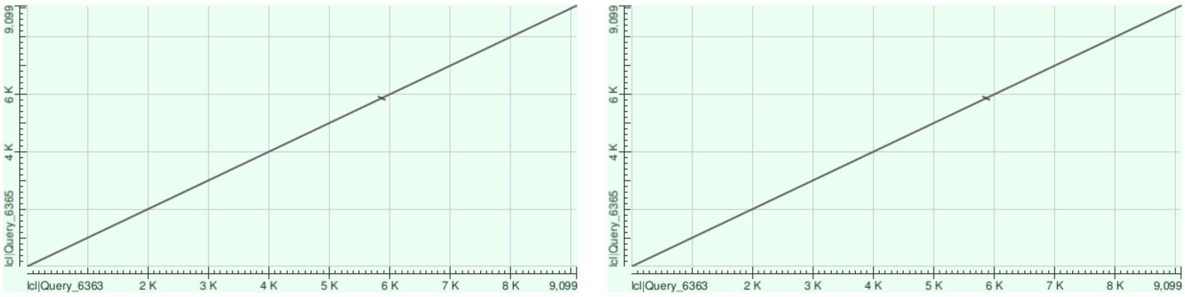

Figure S3 Q: tig00000021 (duplicate to lp17)

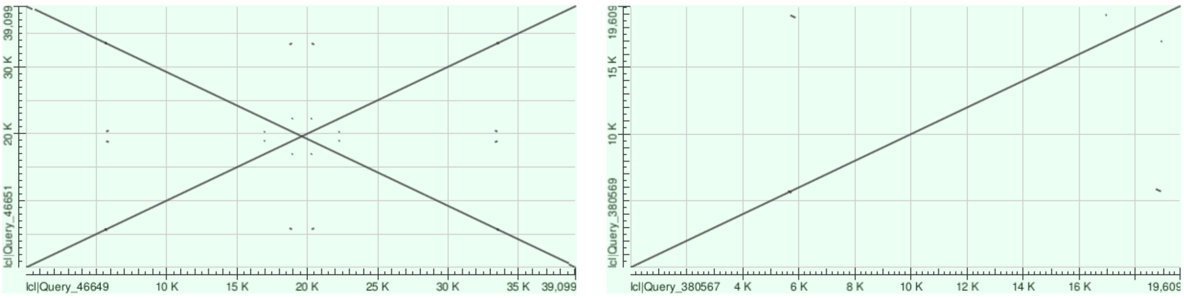

Figure S3 R: tig00000022 (part of lp17)

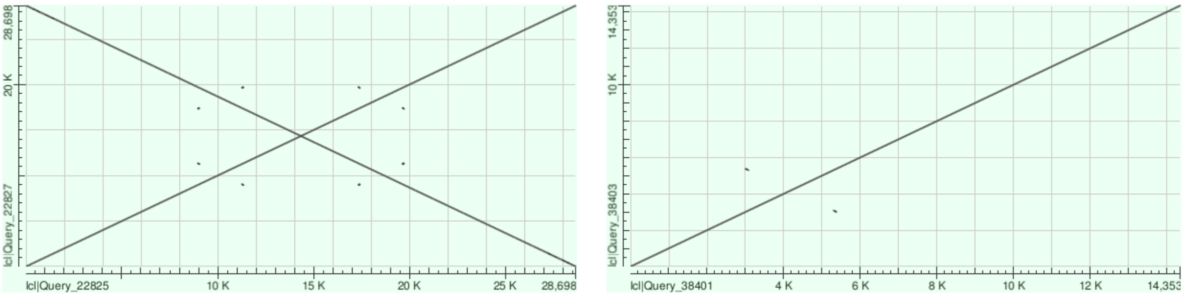

Figure S3 S: tig00000023 (part of lp28-3)

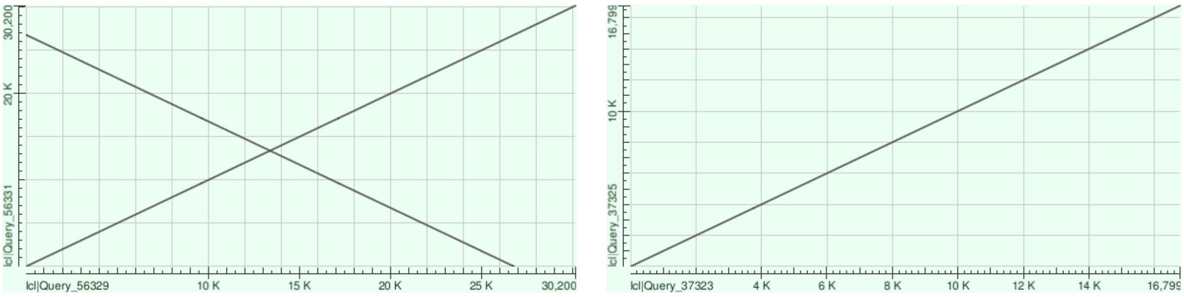

Figure S3 T: tig00000024 (part of lp28-3)

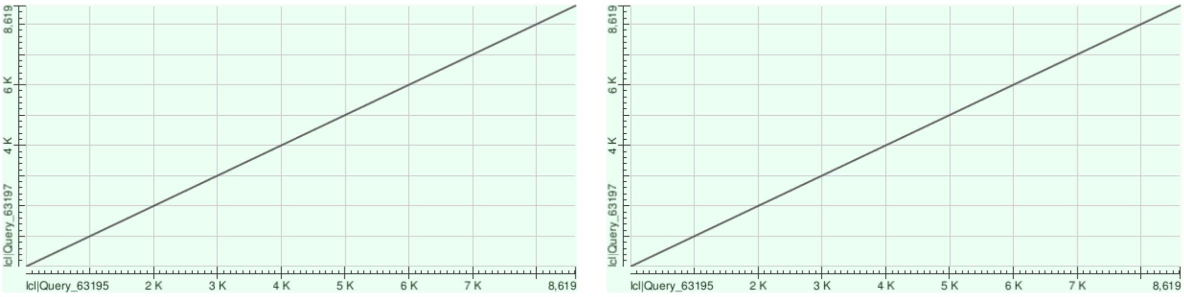

Figure S3 U: tig00000025 (duplicate to lp28-3)

Dot plots B. garinii *PBes, lp32-10, microbial assembler*

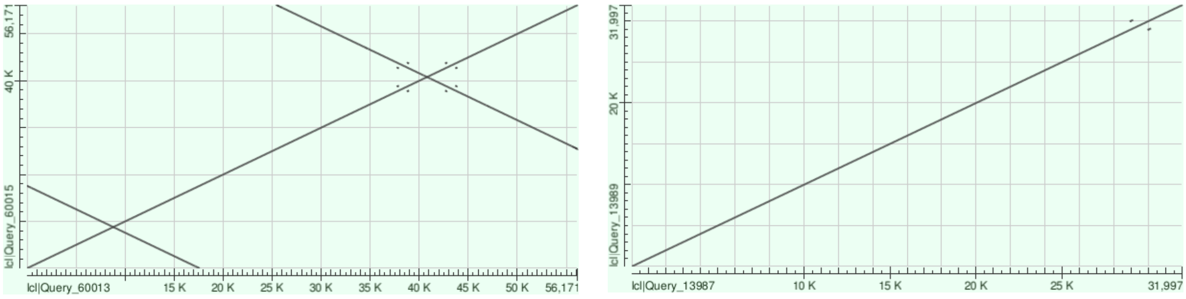

Figure S4: ctg.s2.000002F (untrimmed and trimmed)

Dots plot B. valaisiana *89B13, lp32-7, IPA*

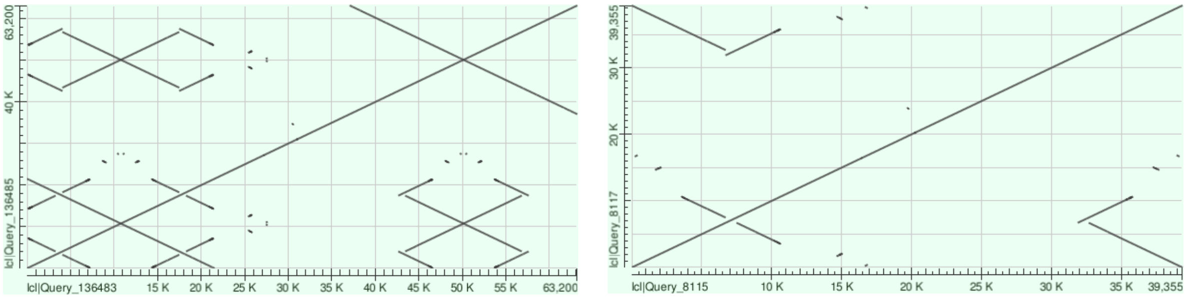

Figure S5: ctg.000004F (untrimmed and trimmed)

Dots plot B. valaisiana *89B13, lp25, microbial_circulomics*

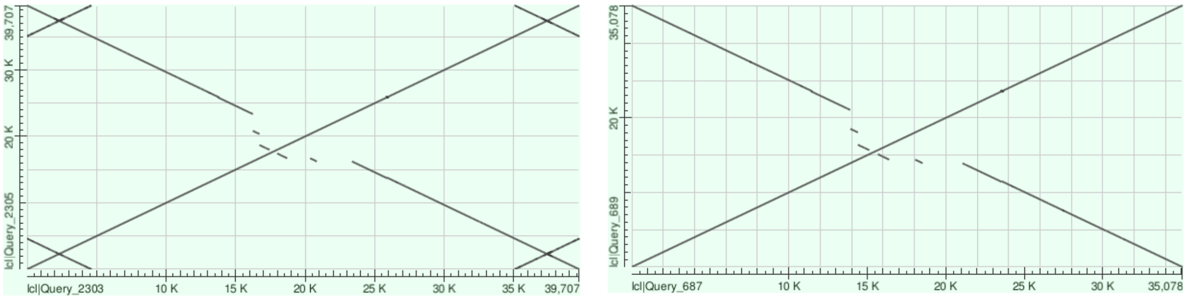

Figure S6: ctg.s2.000020F (untrimmed and trimmed)

Mapping graphs of **B. bavariensis** ***PBaeII***

Figure S7: Mapping graph of PBaeII. PacBio HiFi reads were mapped on the final combined consensus of PBaeII and mapping graphs were generated using CLC genomic workbench 11. The mapping graphs show the coverage depth (y-axis) over the plasmid length (x-axis).

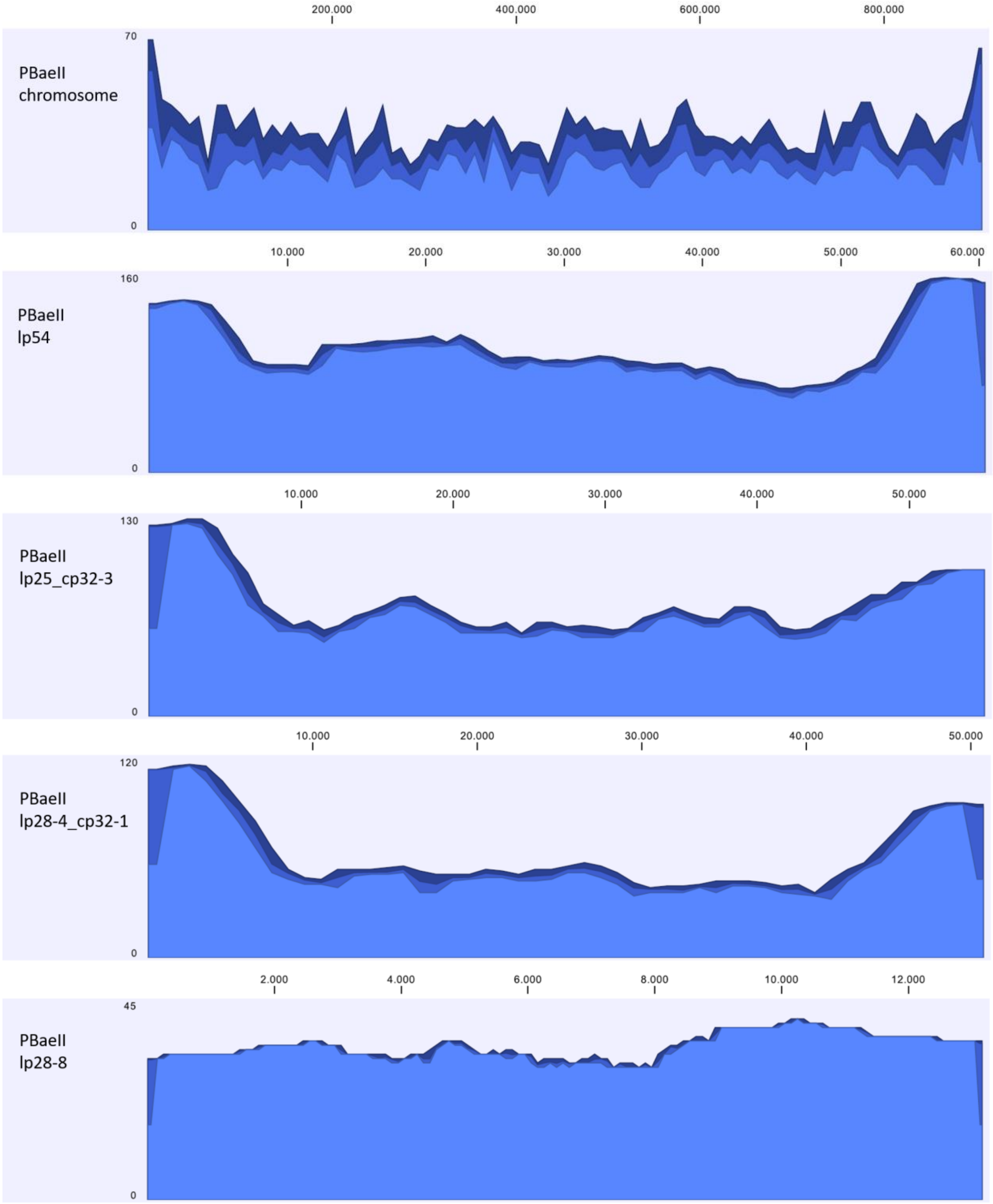

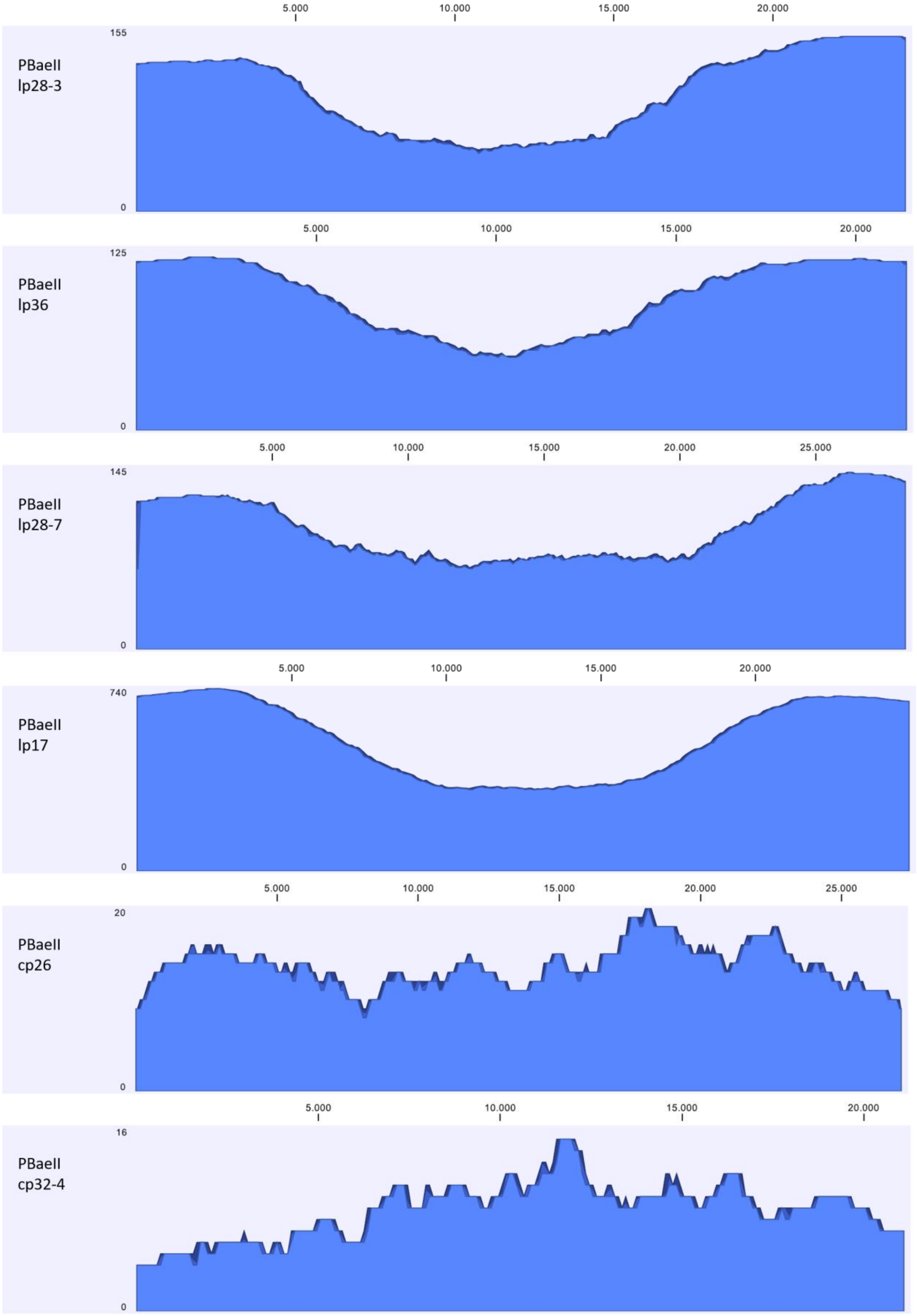

5.000
10.000
15.000
20.000
155
0
PBaeII lp28-3
5.000
10.000
15.000
20.000
125
0
PBaeII lp36
5.000
10.000
15.000
20.000
25.000
145
0
PBaeII lp28-7
5.000
10.000
15.000
20.000
740
0
PBaeII lp17
5.000
10.000
15.000
20.000
25.000
20
0
PBaeII cp26
5.000
10.000
15.000
20.000
16
0
PBaeII cp32-4

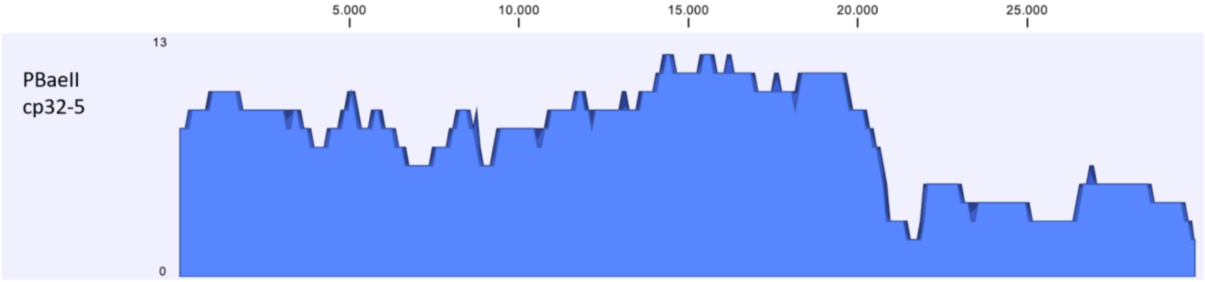

5.000
10.000
15.000
20.000
25.000
13
0
PBaeII
cp32-5
